# DyeVert Contrast Reduction System Use in Patients Undergoing Coronary and/or Peripheral Angiography: A Systematic Literature Review and Meta-Analysis

**DOI:** 10.3389/fmed.2022.841876

**Published:** 2022-04-25

**Authors:** Giuseppe Tarantini, Anand Prasad, Sudhir Rathore, Shweta Bansal, Regine Gottfried, Alexander R. Rosenkranz, Carlo Briguori, Mohsen Yaghoubi, Atefeh Mashayekhi, Mehdi Javanbakht, Eoin Moloney

**Affiliations:** ^1^Department of Cardiac, Thoracic, Vascular Sciences and Public Health, University of Padova, Padua, Italy; ^2^Department of Medicine, Division of Cardiology, University of Texas Health Science Center, San Antonio, TX, United States; ^3^Frimley Health National Health Service (NHS) Foundation Trust, Camberley, United Kingdom; ^4^Department of Medicine, Division of Nephrology, University of Texas Health Science Center, San Antonio, TX, United States; ^5^Clinic for General and Interventional Cardiology and Angiology, Herz- und Diabeteszentrum Nordrhein-Westfalen (NRW), Ruhr-Universität Bochum, Bad Oeynhausen, Germany; ^6^Division of Nephrology, Department of Internal Medicine, Medical University of Graz, Graz, Austria; ^7^Mediterranea Cardiocentro, Naples, Italy; ^8^Mercer University College of Pharmacy, Atlanta, GA, United States; ^9^Optimax Access Ltd., Market Access Consultancy, University of Southampton Science Park, Hampshire, United Kingdom; ^10^Device Access UK Ltd., Market Access Consultancy, University of Southampton Science Park, Hampshire, United Kingdom

**Keywords:** DyeVert System, acute kidney injury, contrast media, contrast induced nephropathy, coronary angiography, percutaneous coronary intervention, systematic review, meta-analysis

## Abstract

**Background:**

Contrast-associated acute kidney injury (CA-AKI) is an important adverse effect associated with injecting iodinated intra-arterial contrast media (CM) during coronary angiography. The DyeVert™ Contrast Reduction System is a medical device intended to reduce the intra-arterial CM volume (CMV) administered. The aim of this study was to assess DyeVert System clinical effectiveness and safety by implementing a systematic review and meta-analysis of existing evidence.

**Methods:**

Systematic electronic literature searches were conducted in MEDLINE, Embase, the Cochrane Database of Systematic Reviews, ClinicalTrials.gov, and the International Clinical Trials Registry Platform database. Relevant data were extracted from included studies and meta-analyses were performed to synthesize evidence across studies.

**Results:**

The review included 17 eligible studies involving 1,731 DyeVert System cases and 1,387 control cases (without the use of DyeVert). Meta-analyses demonstrated use of the DyeVert System reduced CMV delivered to the patient by 39.27% (95% CI, 36.10–42.48%, *P* < 0.001), reduced CMV/baseline renal function ratios (Hedges’s g, −0.56; 95% CI, −0.70 to −0.42, *P* < 0.001) and percentage of cases exceeding the maximum CMV threshold (risk difference −0.31, 95% CI, −0.48 to −0.13, *P* < 0.001) while maintaining adequate image quality in 98% of cases. DyeVert System cases demonstrated lower CA-AKI incidence vs. controls (absolute risk reduction 5.00% (95% CI, 0.40–9.80%; *P* = 0.03), relative risk 0.60 (95% CI, 0.40–0.90; *P* = 0.01) with a pooled estimate of the number needed to treat with the DyeVert System to avoid 1 CA-AKI event of 20.

**Conclusion:**

DyeVert System use significantly reduces CMV delivered to the patient, CMV/baseline renal function ratios, and CA-AKI incidence while maintaining image quality. Accordingly, the device may serve as an adjunctive, procedure-based strategy to prevent CA-AKI. Future multi-center studies are needed to further assess effects of minimizing CMV on endpoints such as CA-AKI prevention, incidence of adverse cardiac and renal events, and health care costs.

## Introduction

Intra-arterial injection of contrast media (CM) is used to enhance image quality, allowing radiologists and clinicians to better distinguish between various body tissues when performing techniques such as coronary angiography and percutaneous coronary intervention (PCI). While beneficial for clinical interpretation and diagnosis, use of CM may result in adverse effects (1). One of the most important adverse effects associated with CM is contrast-associated acute kidney injury (CA-AKI) with incidence rates between 3 and 37% (2–6).

Currently, there is no treatment for CA-AKI. Therefore, clinical attention to CA-AKI focuses on prevention, especially for high-risk patients (7). Patients with comorbidities such as advanced age, co-existing chronic kidney disease (CKD), heart failure, anemia, and/or diabetes are at increased risk for CA-AKI, as are patients undergoing procedures with acute clinical presentation or high complexity as shown by previous CA-AKI risk prediction models (8). Higher contrast media volume (CMV) and, particularly, high CMV relative to individual patient baseline renal function are significantly associated with increased CA-AKI incidence (9–12). Clinical practice guidelines and recommendations for CA-AKI prevention have emphasized procedure-based strategies, including pre-procedural patient risk assessment, discontinuing potentially nephrotoxic medications prior to contrast administration, adequate volume expansion, and monitoring and minimizing CMV administered, particularly in patients with CKD (13–17). Yet despite a decade of clinical practice, these recommendations remain incompletely and inconsistently implemented (18, 19).

The FDA-cleared and CE-marked DyeVert™ Contrast Reduction System (Osprey Medical, Minnetonka, Minnesota, United States) is intended to reduce the CMV administered during diagnostic and interventional angiographic procedures while maintaining image quality. The DyeVert, DyeVert Plus, and DyeVert Plus EZ Contrast Reduction Systems are compatible with manual contrast injection, while the DyeVert Power XT Contrast Reduction System is compatible with automated contrast injection. By enabling accurate, real-time monitoring of CMV administered relative to a pre-determined maximum CMV threshold entered into the display, the DyeVert System provides clinicians in the catheterization laboratory with continuous data on CM use to facilitate intraprocedural decision-making. Additionally, the device diverts excess CM not required to accomplish procedural objectives, resulting in reduced contrast load to the patient.

We aimed to assess the effect of the DyeVert System in diagnostic coronary angiography and/or PCI procedures by pooling all relevant studies in a systematic review and performing meta-analyses to quantitatively synthesize evidence on outcomes.

## Methods

We performed a systematic literature review to identify studies reporting the clinical effectiveness and/or safety of the DyeVert System. The literature review was conducted according to methodological guidance from the Center for Reviews and Dissemination on best practices for conducting systematic reviews in health care (20). We followed the Preferred Reporting Items for Systematic Reviews and Meta-Analyses (PRISMA) approach to report findings of the review and meta-analyses (21). The authors had full access to all data included in the study.

### Search Strategy

Systematic electronic searches were conducted July 2021 in MEDLINE, Embase, the Cochrane Database of Systematic Reviews, the ClinicalTrials.gov database, and the International Clinical Trials Registry Platform database. Additional Internet searches were performed to identify any further publications of interest. Supplementary Material provide search strategy details (Supplementary Tables 1–5). Records meeting the search criteria were downloaded from databases and imported into Microsoft^®^ Excel^®^ software, where duplicate records were removed.

### Study Selection

**FIGURE 1:**
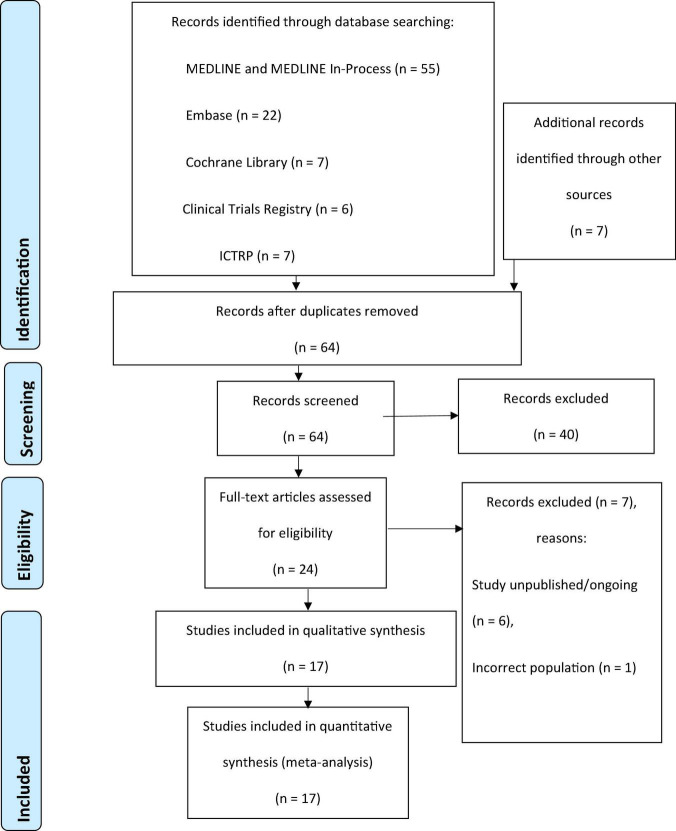
PRISMA flow diagram for the systematic review. ICTRP, International Clinical Trials Registry Platform; PRISMA, Preferred Reporting Items for Systematic Reviews and Meta-Analyses.

Studies selected for inclusion in the review met these selection criteria: (1) patients were adults undergoing diagnostic coronary angiography, PCI, and/or a peripheral intervention procedure using intra-arterial injection of CM and (2) DyeVert, DyeVert Plus, DyeVert Plus EZ, or DyeVert Power XT systems were used as an intervention. Studies presented in a language other than English and studies or publications representing economic analyses, editorials, reviews, book chapters, or letters were excluded from this review and meta-analysis. Two reviewers systematically screened titles and abstracts. A third reviewer resolved any disagreements regarding potential exclusions. Remaining studies underwent full-text screening for eligibility. References of eligible studies were searched manually to identify additional relevant studies, although no further studies were found. The study selection process is depicted in a PRISMA diagram in Figure 1 (21).

### Data Extraction

All eligible studies were reviewed, and relevant data were extracted by 1 reviewer; another independent reviewer performed quality control. Disagreements between the reviewers regarding extracted data were resolved by discussion, and a third reviewer participated as needed to reach consensus. Extracted data included study design, location, and setting; type of intervention and comparators; characteristics of the patient population (including details related to patient follow-up and withdrawal); main outcomes; and results reported (including clinical effectiveness and safety of the intervention).

### Outcomes of Interest

Outcomes of interest included CMV delivered to the patient, CMV diverted with the DyeVert System, CMV threshold management, CMV/baseline renal function ratios, image quality, CA-AKI incidence, and DyeVert System-related adverse events.

### Statistical Analysis

We performed meta-analyses to quantitatively synthesize the findings related to CMV use, CMV threshold management, CMV/baseline renal function ratios, image quality and CA-AKI incidence. The number of studies included in each meta-analysis varied depending on outcomes reported in each study. Fixed- and random-effects analyses were performed. The Mantel-Haenszel method was used to calculate the fixed-effects estimate, with inverse-variance weighting using the DerSimonian and Laird method used to account for heterogeneity in the random-effects model (22, 23). Cochran’s Q statistic and the *I*^2^ statistic were calculated to test for heterogeneity across studies, with an *I*^2^ statistic > 50% indicating a moderate to strong (> 75%) presence of heterogeneity (24, 25). Egger’s test and Begg’s test statistics were used to evaluate potential publication bias (26). Forest plots were produced to show pooled estimates. All *p*-values were 2-tailed with statistical significance set at < 0.05. Computations were performed using StataCorp and MedCalc.

### Quality Assessment

All included studies were quality-assessed using an appropriate critical appraisal tool.

## Results

### Systematic Review

The initial systematic, electronic search identified 97 studies, and Supplementary Internet searches identified 7 additional potentially relevant articles. From these 104 studies, 40 duplicates were removed. The remaining 64 studies were screened for eligibility, of which 40 studies were excluded based on screening of titles and abstracts. Of 24 studies that underwent full-text screening, 7 were deemed ineligible based on exclusion criteria. The remaining 17 studies were included in the qualitative review and quantitative meta-analysis (27, 28). Results of the study selection process are depicted in Figure 1.

Table 1 summarizes the 17 included studies from 8 full text articles and 9 abstracts; all studies were published or presented (in the case of poster abstracts) from 2017 through 2020. Collectively, the studies involved 1,731 DyeVert System cases and 1,387 control cases. All 17 studies referred to use of the DyeVert System as an intervention implemented in combination with CM injection systems used routinely at each site (DyeVert group). Fifteen studies involved the use of manual CM injection systems (Table 1). Two studies reported use of the DyeVert System with an automated CM injection system, of which Bruno et al. was a sub-set of Amoroso et al. data and reported the author’s single-center experience (28, 43). Nine studies included a control group composed of cases using routine CM injection systems and imaging practices without the DyeVert System (control group). Eight studies did not involve a control group. Most publications include real-world data reflecting the modern health care system, current practices for care delivery, and complexity of patient demographics. Kutschman et al. published two reports of a 6-month hospital quality improvement project—one involving the overall population (33) and one focused on a subgroup with CKD (32).

**TABLE 1:** Summary of studies included in systematic review and meta-analysis.

References	Publication type	Location	Design	Number of hospitals	No. patients enrolled by study arm	
					DyeVert System	Control
Desch et al. (27)	Manuscript	Germany	P, RCT	1	(DyeVert) 48	(Manual injection) 48
Bath et al. (29)	Abstract	United States	P, RCT	1	(DyeVert Plus) 49	(Manual injection) 59
Briguori et al. (30)	Manuscript	Italy	R, O, PMC	1	(DyeVert Plus EZ) 90	(Manual injection) 90
Sattar et al. (31)	Abstract	United States	R, O	1	(DyeVert Plus) 41	(Manual injection) 68
Kutschman (32)	Abstract	United States	R, O	1	(DyeVert Plus & DyeVert Plus EZ) 128	(Manual injection 78
Kutschman et al. (33)	Abstract	United States	R, O	1	(DyeVert Plus & DyeVert Plus EZ) 258	(Manual injection) 243
Bunney et al. (34)	Abstract	United States	R, O	1	(DyeVert Plus & DyeVert Plus EZ) 29	(Manual injection) 770
Tajti et al. (35)	Manuscript	United States	R, O	1	(DyeVert Plus) 40	(Manual injection) 94
Zimin et al. (36)	Manuscript	United States	P, O	1	(DyeVert Plus EZ) 16	(Manual injection with OCT imaging) 15
Sapontis et al. (37)	Manuscript	Germany, Australia	P, O	2	(DyeVert) 44	None
Corcione et al. (38)	Manuscript	Italy	R, O	1	(DyeVert Plus) 10	None
Gurm et al. (39)	Manuscript	United States	P, O	8	(DyeVert Plus) 114	None
Turner and Tucker (40)	Abstract	United States	R, O	1	(DyeVert Plus & DyeVert Plus EZ) 536	None
Cameron and Espinosa (41)	Abstract	United States	R, O	1	(DyeVert Plus & DyeVert Plus EZ) 423	None
Rao (42)	Abstract	United States	R, O	1	(DyeVert Plus) 7	None
Amoroso et al. (43)	Abstract	Netherlands, Germany, United Kingdom	R, O	3	(DyeVert Power XT) 26	None
Bruno et al. (28)	Manuscript	Germany	R, O	1	(DyeVert Power XT) 9	None

*O, observational; OCT, optical coherence tomography; P, prospective; PMC, propensity-matched control; R, retrospective; RCT, randomized controlled trial.*

Table 2 summarizes patient baseline characteristics. Patients were predominantly male with advanced age and many studies included patients with co-morbidities such as CKD, hypertension, diabetes, heart failure, peripheral arterial disease, prior myocardial infarction, and prior PCI. Table 3 summarizes procedure characteristics. Cases involving radial as well as femoral access are represented. Most procedures involved PCI (either exclusively or combined with CA). Four studies involved chronic total occlusions in which one or two contrast injection lines may be used; and therefore, one or two DyeVert Systems may be used depending on the technique employed. Two studies involved peripheral vascular interventions. Iso-osmolar and low osmolar contrast agents were used. Supplementary Material contain additional reported baseline and procedure characteristics (Supplementary Table 6) and CA-AKI prevention strategies (Supplementary Table 7).

**TABLE 2:** Baseline clinical characteristics across included studies.

References	Study arm	Age (y)	Gender (male, %)	eGFR (mL/min/1.73 m^2^)	SCr (mg/dL)	HTN (%)	DM (%)	CKD (%)	HF (%)	Prior PCI (%)
Desch et al. (27)	DyeVert	69 ± 14	58	NR	NR	73	13	69	42	23
	Control	66 ± 13	58	NR	NR	71	17	77	40	27
Bath et al. (29)	DyeVert	NR	NR	NR	NR	NR	NR	100	NR	NR
	Control	NR	NR	NR	NR	NR	NR	100	NR	NR
Briguori et al. (30)	DyeVert	63 ± 13	71	74 ± 26	1.0 (0.8–1.1)	61	22	30	NR	NR
	Control	64 ± 13	77	79 ± 28	1.0 (0.8–1.2)	62	18	23	NR	NR
Sattar et al. (31)	DyeVert	69	41	43.6	1.6	90	54	100	NR	NR
	Control	71	65	47.7	1.5	93	51	100	NR	NR
Kutschman (32)	Overall	69 ± 11	57	43 ± 13	NR	NR	NR	100	NR	NR
Kutschman et al. (33)	Overall	64 ± 32	63	64 ± 32	1.6 ± 1.6	82	55	33	23	27
Bunney et al. (34)	DyeVert	63	NR	NR	NR	NR	72	55	28	NR
	Control	61	NR	NR	NR	NR	48	24	15	NR
Tajti et al. (35)	DyeVert	68 ± 9	82	72 (55–83)	1.1 (0.9–1.2)	90	49	NR	21	72
	Control	66 ± 12	78	77 (57–89)	1.0 (0.9–1.2)	83	42	NR	20	63
Zimin et al. (36)	DyeVert	67 ± 11	79	71 ± 20	1.0 ± 0.3	86	50	7	14	57
Sapontis et al. (37)	DyeVert	69 ± 11	68	NR	NR	75	34	43	18	36
Corcione et al. (38)	DyeVert	66 ± 12	80	81 ± 20	1.0 ± 0.2	NR	NR	30	NR	NR
Gurm et al. (39)	DyeVert	72 ± 9	72	43 ± 11	1.6 ± 0.5	96	53	100	47	53
Turner and Tucker (40)	Overall	NR	NR	NR	NR	NR	NR	NR	NR	NR
Cameron and Espinosa (41)	Overall	NR	NR	NR	NR	NR	NR	24	NR	NR
Rao (42)	DyeVert	66 ± 13	43	46 ± 29	1.6 ± 0.6	71	86	86	57	14
Amoroso et al. (43)	DyeVert	NR	NR	NR	NR	NR	NR	NR	NR	NR
Bruno et al. (28)	DyeVert	71 ± 10	56	72 ± 9	1.2 ± 0.4	78	44	NR	67	NR

*Data presented are mean ± standard deviation, median (IQR), or %.*

*CKD, chronic kidney disease; DM, diabetes mellitus; eGFR, estimated glomerular filtration rate; HF, heart failure; HTN, hypertension; NR, not reported; PCI, percutaneous coronary intervention; SCr, serum creatinine.*

**TABLE 3:** Procedure characteristics across included studies.

References	Study arm	Procedure type					Access		CM type
		CA only (%)	CA + PCI (%)	PCI only (%)	CTO (%)	PVI (%)	Radial (%)	Femoral (%)	
Desch et al. (27)	DyeVert	100	0	0	0	0	48	NR	Iomeprol (Imeron 350) Iomeprol (Imeron 350)
	Control	100	0	0	0	0	46	NR	
Bath et al. (29)	DyeVert	100	0	0	0	0	NR	NR	NR
	Control	100	0	0	0	0	0	NR	NR
Briguori et al. (30)	DyeVert	1	99*		NR	0	97	NR	Iobitridol (Xenetix 350)
	Control	2	98*		NR	0	99	NR	Iobitridol (Xenetix 350)
Sattar et al. (31)	DyeVert	0	100*		NR	0	NR	NR	NR
	Control	0	100*		NR	0	NR	NR	NR
Kutschman (32)	Overall	37	63*		10	0	NR	NR	NR
Kutschman et al. (33)	Overall	31	69*		9	0	NR	NR	NR
Bunney et al. (34)	DyeVert	0	100*		NR	0	NR	NR	NR
	Control	0	100*		NR	0	NR	NR	NR
Tajti et al. (35)	DyeVert	0	100*		100	0	50	95	Iodixanol (Visipaque) 79.5%; iohexol (Omnipaque) 20.5%
	Control	0	100*		100	0	19	92	NR
Zimin et al. (36)	DyeVert	0	87	13	0	0	47	53	Iopamidol (Isovue 370)
	Control	7	93	0	0	0	0	100	Iopamidol (Isovue 370)
Sapontis et al. (37)	DyeVert	77	16	7	NR	0	NR	NR	Iodixanol 320 52%; iohexol 350 46%; other 2%
Corcione et al. (38)	DyeVert	50	10	20	0	20	70	30	Iohexol (Omnipaque 350) 50%; iopromide (Ultravist 370) 30%; ioversol (Optiray 350) 20%
Gurm et al. (39)	DyeVert	65	26	9	0	0	37	63	Iodixanol (Visipaque 320) 55%; iohexol (Omnipaque 350) 18%; iohexol (Omnipaque 300) 14%; iopamidol (Isovue) 7%; iodixanol (Visipaque 270) 6%
Turner and Tucker (40)	Overall	0	100*		NR	0	NR	NR	NR
Cameron and Espinosa (41)	Overall	57	43*		NR	0	NR	NR	NR
Rao (42)	DyeVert	0	0	0	43	100	0	100	Iodixanol (Visipaque 320)
Amoroso et al. (43)	DyeVert	54	46*		0	0	85	NR	Accupaque 350 35%; Niopam 300 27%; iodixanol (Visipaque 270) 38%
Bruno et al. (28)	DyeVert	78	22	0	0	0	NR	NR	Accupaque 350

*PCI proportion with or without a diagnostic coronary angiography component was not reported.

*Data presented are %.*

*CA, diagnostic coronary angiography; CM, contrast media; CTO, chronic total occlusion; NR, not reported; PCI, percutaneous coronary intervention; PVI, peripheral vascular intervention.*

### Outcomes

Table 4 summarizes CMV use and threshold management. Mean CMV “attempted,” defined as the CMV that would have been delivered if the DyeVert System had not been used, calculated by summing the actual CMV delivered to the patient and the CMV diverted (or “saved”) by the DyeVert System, ranged from 112 ml in a study involving 65% diagnostic coronary angiography cases to 342 ml in a PCI study. In cases involving DyeVert System use, the mean CMV diverted ranged from 42 to 126 ml, which equated to a mean 34–47% of the attempted CMV being diverted with the DyeVert System. Overall mean CMV delivered to the patient ranged from 37 to 216 ml in DyeVert group cases and 63–250 ml in control group cases.

**TABLE 4:** Contrast media volume and threshold management.

References	Study arm	CMV attempted (mL)	CMV diverted by the DyeVert System (mL)	CMV delivered to patient (mL)	% of the Attempted CMV diverted by the DyeVert System (%)	% Cases with actual CMV ≤ CMV threshold	% Cases with attempted CMV ≤ CMV threshold
Desch et al. (27)	DyeVert	NR	NR	37 ± 11	NR	NR	NR
	Control	NA	NA	63 ± 13	NA	NR	NA
Bath et al. (29)	DyeVert	NR	NR	63 ± 10	35 ± 3	NR	NR
	Control	NA	NA	88 ± 11	NA	NR	NA
Briguori et al. (30)	DyeVert	NR	NR	99 ± 50	38 ± 13	NR	NR
	Control	NA	NA	130 ± 50	NA	NR	NA
Sattar et al. (31)	DyeVert	NR	NR	128	NR	NR	NR
	Control	NA	NA	155	NA	NR	NA
Kutschman (32)	DyeVert	NR	NR	Overall 103 ± 61	41 ± 8	NR	NR
	Control	NA	NA		NA	NR	NA
Kutschman et al. (33)	DyeVert	NR	58	104 ± 60	40	86	67
	Control	NA	NA	126 ± 81	NA	75	NA
Bunney et al. (34)	DyeVert	NR	NR	194	NR	NR	NR
	Control	NA	NA	192	NA	NR	NA
Tajti et al. (35)	DyeVert	NR	NR	200 (153–256)	NR	NR	NR
	Control	NA	NA	250 (170–303)	NA	NR	NA
Zimin et al. (36)	DyeVert	342 ± 130	126 ± 47	216 ± 89	38 ± 5	93	13
Sapontis et al. (37)	DyeVert	173 ± 117	84 ± 66	89 ± 57	47 ± 9	NR	NR
Corcione et al. (38)	DyeVert	136 ± 74	56 ± 32	80 ± 49	42 ± 7	NR	NR
Gurm et al. (39)	DyeVert	112 ± 85	NR	67 ± 51	40 ± 9	87	62
Turner and Tucker (40)	DyeVert	137 ± 82	42 ± 28	95 ± 61	NR	82	62
Cameron and Espinosa (41)	DyeVert	144 ± 79	53 ± 28	91 ± 55	38 ± 8	84	58
Rao (42)	DyeVert	NR	NR	50 ± 23	NR	86	NR
Amoroso et al. (43)	DyeVert	NR	NR	88 ± 52	34 ± 6	NR	NR
Bruno et al. (28)	DyeVert	129	NR	81 ± 54	39	NR	NR

*Data presented are mean, mean ± standard deviation, median (IQR), or %.*

*CMV, contrast media volume; NA, not applicable; NR, not reported.*

Because of the CMV diverted by the DyeVert System, a larger proportion of cases delivered CMV to the patient at or below the maximum CMV dose threshold prespecified by the physician and resulted in lower mean CMV/baseline renal function ratios as well as a larger proportion of cases achieving lower CMV/baseline renal function ratios (Table 4 and Supplementary Table 8, respectively).

Image quality was assessed during DyeVert System use in 9 studies by the physician at the time of the procedure (Supplementary Table 9) and was reported to be adequate in 96–100% of cases. Two of these studies additionally involved the use of independent reviewers to assess image quality of DyeVert group cases compared to control group cases and reported non-inferior image quality (27, 36).

CA-AKI incidence was reported in 11 publications (Supplementary Table 10). CA-AKI definitions were not reported in 2 studies (35, 38); however, 9 studies did report the definition used, which ranged from worsening renal function to serum creatinine (SCr) increase ≥ 0.3 mg/dL or 50% within 48 h of the procedure. In studies involving a control group, observed CA-AKI incidence was lower in DyeVert group cases. Briguori et al. involved the use of propensity matching of DyeVert and control group in acute coronary syndrome patients. Tucker et al. and Cameron et al. reported overall CA-AKI incidence from hospital quality improvement efforts demonstrating a meaningful decrease in CA-AKI between the initial and final follow-up intervals. Kutschman et al. reported an observed 33% relative reduction in CA-AKI in the DyeVert group compared to the control group in the overall population included in a hospital quality improvement program and a 57% relative reduction in the CKD subgroup. Tajti et al. studied DyeVert System use in chronic total occlusion cases and additionally reported a post-procedure CA-AKI incidence though the CA-AKI definition was not reported.

Nine studies reported on adverse events and no DyeVert System-related adverse events were reported (27, 28, 30, 35–39, 42). Corcione et al. described a case of contrast-induced nephropathy in a patient who underwent a combined carotid angiography and angioplasty and experienced elevated serum creatinine levels that returned to baseline 3 days after the procedure; the authors noted the event was not device-related (38). Briguori et al. and Tajti et al. further reported frequencies of reported adverse events were similar in the DyeVert and control Groups, with no adverse events identified as being related to use of the DyeVert System (30, 35).

### Meta-Analysis

#### Contrast Media Volume Use

**FIGURE 2:**
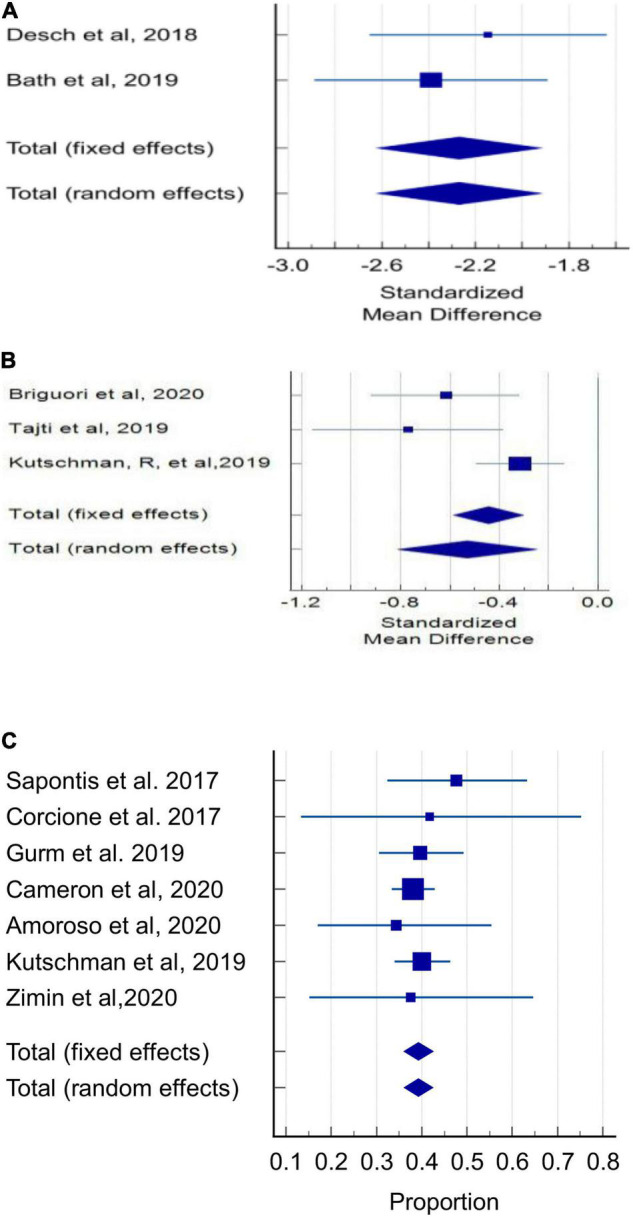
Contrast media volume use. **(A)** Forest plot of mean difference in absolute contrast volume (mL) in randomized controlled trials. **(B)** Forest plot of mean difference in absolute contrast volume (mL) in observational studies. **(C)** Forest plot of proportion of attempted CMV that was diverted (%) in observational studies.

The pooled estimate of the standardized mean difference in absolute CMV (mL) delivered to the patient between the DyeVert group and control group in 2 randomized controlled trials was -2.27 (95% CI, −2.62 to 1.92; *P* < 0.001) (Figure 2A and Supplementary Table 11). In this meta-analysis, there was no evidence of heterogeneity (*I*^2^ = 0%) and Egger’s test was significant for publication bias (*P* < 0.0001). In 3 retrospective, observational, 2-arm studies, the pooled estimate of the standardized mean difference in absolute CMV (mL) between the DyeVert group and control group was −0.53 (95% CI, −0.81 to −0.25; P < 0.0.001) (Figure 2B and Supplementary Table 12). Two studies that reported mean differences without standard deviations were excluded from this meta-analysis (31, 34). This analysis showed moderate evidence of heterogeneity (*I*^2^ = 67%), and Egger’ test was significant for publication bias (*P* = 0.049). These results indicate DyeVert System use resulted in a significant decrease in CMV relative to the control group.

Among 7 observational studies, in the DyeVert group, the pooled estimate of the percentage of the total attempted CMV diverted by the DyeVert System was 39.27% (95% CI, 36.10–42.48%; *P* < 0.01, Figure 2C and Supplementary Table 13A) for all studies and 39.47% (95% CI, 36.24–42.73%; *P* < 0.01) for studies using manual CM injection systems (Supplementary Figure 1 and Supplementary Table 13B). In both analyses, there was no evidence of heterogeneity (*I*^2^ = 0%) and Egger’s test was not significant for publication bias (*P* = 0.48 and *P* = 0.24, respectively).

#### Contrast Media Volume Threshold Management

**FIGURE 3:**
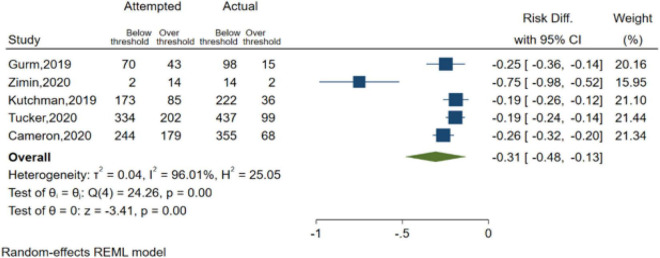
Contrast media volume threshold management in the DyeVert group.

In DyeVert group cases, at the beginning of each case, the physician enters the predefined maximum CMV threshold into the display. This analysis compared the predetermined CMV threshold with the CMV attempted and CMV delivered to the patient across 5 studies (Figure 3). Results indicated use of the DyeVert System resulted in significantly reduced risk of a patient receiving a CMV that exceeded their maximum CMV threshold (risk difference, −0.31; 95% CI, −0.48 to −0.13, *P* < 0.001).

#### Contrast Media Volume/Baseline Renal Function Ratios

**FIGURE 4:**
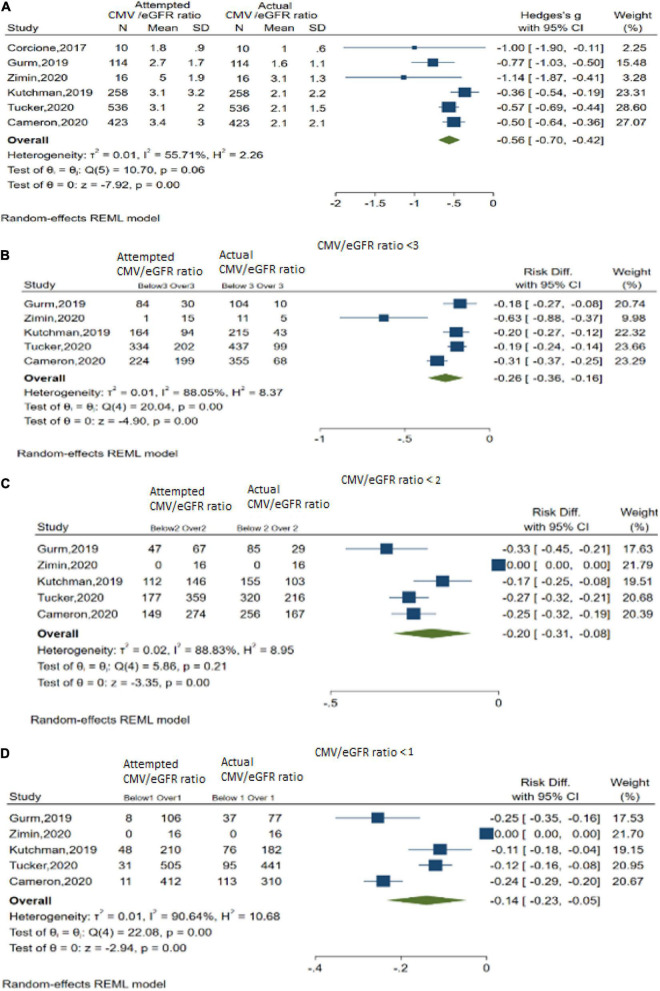
Contrast media volume/baseline renal function ratios. **(A)** Overall meta-analysis across 6 studies. **(B)** Meta-analysis for CMV/eGFR ratio < 3. **(C)** Meta-analysis for CMV/eGFR ratio < 2. **(D)** Meta-analysis for CMV/eGFR ratio < 1.

In DyeVert group cases, 6 studies explored the actual CMV/estimated glomerular filtration rate (eGFR) ratio vs. attempted CMV/eGFR ratio (Figures 4A–D). Results indicated use of the DyeVert System resulted in significantly reduced actual CMV/eGFR compared with the attempted CMV/eGFR (Hedges’s g, −0.56; 95% CI, −0.70 to −0.42, *P* < 0.001) (Figure 4A). Five studies reported additional outcomes by 3 different CMV/eGFR ratios. Results indicate DyeVert System use significantly reduced the risk of receiving a CMV exceeding each of the 3 CMV/eGFR ratio subgroups (Figures 4B–D). All 3 analyses showed strong evidence of heterogeneity (*I*^2^ ≥ 88%).

#### Image Quality

**FIGURE 5:**
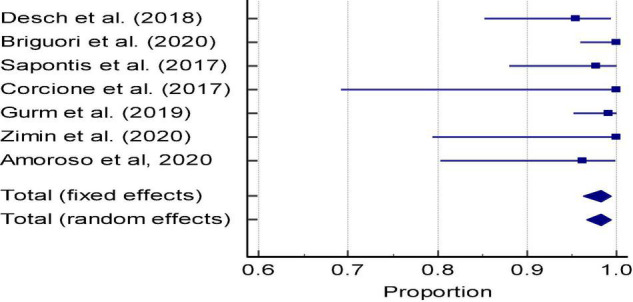
Meta-analysis: Forest plot of image quality in the DyeVert group (Proportion of cases with adequate image quality per physician assessment).

In DyeVert group cases, 7 studies reported image quality based on clinician feedback during the case (Figure 5 and Supplementary Table 14). The pooled estimate of percent cases in which clinicians reported adequate fluoroscopic image quality during DyeVert System use was 98.21% (95% CI, 96.54–99.34%). This analysis demonstrated low evidence of heterogeneity (*I*^2^ = 1.74%), and Egger’s test was not significant for publication bias (*P* = 0.27). Results indicate the overall image quality was adequate in a majority of cases.

#### Contrast-Associated Acute Kidney Injury Incidence

Five two-arm studies assessed CA-AKI incidence (Table 5). Pooled incidence CA-AKI in the DyeVert group was 7.30% (95% CI, 5.11–9.85%). There was no evidence of heterogeneity in this analysis (*I*^2^ = 0%), and Egger’s test was not significant for publication bias (*P* = 0.93; Supplementary Table 15A). Pooled incidence of CA-AKI in the control group was 10.65% (95% CI, 6.60–15.52%). The control group analysis showed strong evidence of heterogeneity based on the Q statistic (*P* < 0.001) and *I*^2^ = 78.54%; Egger’s test was non-significant for publication bias (*P* = 0.71; Supplementary Table 16A).

**TABLE 5:** Contrast-associated acute kidney injury.

References	CA-AKI definition	Proportion of cases with CA-AKI (%)		CA-AKI Absolute risk reduction (%) (95% CI)	Relative risk of a CA-AKI event (95% CI)
		DyeVert group (95% CI)	Control group (95% CI)		
Briguori et al. (30)	SCr ↑ ≥ 0.3 mg/dL within 72 h	8.00 (3.33–15.65)	19.00 (11.50–28.64)	11.00 (−3.73 to 25.73)	0.42 (0.18–0.95)
Kutschman et al. (33)	SCr ↑ ≥ 0.3 mg/dL or 50% within 48 h	6.90 (4.13–10.71)	10.30 (6.78–14.83)	3.00 (−5.78 to 11.78)	0.67 (0.37–1.20)
Sattar et al. (31)	SCr ↑ ≥ 0.3 mg/dL or 50%	12.20 (4.08–26.20)	16.21 (8.38–27.14)	4.00 (−15.04 to 23.04)	0.75 (0.28–2.01)
Bunney et al. (34)	SCr ↑ ≥ 0.3 mg/dL or 50% within 48 h	3.45 (0.09–17.76)	9.35 (7.39–11.63)	6.00 (−0.95 to 12.95)	0.37 (0.05–2.56)
Tajti et al. (35)	NR	2.56 (0.06–13.48)	2.20 (0.27–7.72)	−0.4.00 (−2.00 to 2.20)	1.17 (0.11–12.5)
	Pooled	7.30 (5.11–9.85)	10.65 (6.60–15.52)	5.00 (0.40 to 9.80)	0.60 (0.40–0.90)

*CA-AKI, contrast-associated acute kidney injury; CI, confidence interval; NR, not reported; SCr, serum creatinine.*

Pooled estimate of the absolute risk reduction for CA-AKI associated DyeVert System use was 5.00% (95% CI, 0.40–9.80%; *P* = 0.03) and the pooled relative risk of CA-AKI was 0.60 (95% CI, 0.40–0.90; *P* = 0.01) (Supplementary Table 17A). There was no evidence of heterogeneity in the analysis of absolute risk reduction (*I*^2^ = 0%; Supplementary Table 17A). The pooled estimate of the number of patients needed to be treated to avoid 1 CA-AKI event was 20 (Supplementary Table 17A).

Tajti et al. was different from the other four studies in that it was performed exclusively in chronic total occlusion cases and did not report the CA-AKI definition used. The meta-analysis also was repeated with this study excluded and overall results were similar (Supplementary Tables 15B, 16B, 17B).

See Supplementary Material for additional tables and figures.

### Quality Assessment

Results of the quality assessment of included studies are presented in Supplementary Tables 18–34.

## Discussion

This is the first systematic review and meta-analysis of the DyeVert System. This review included data from 17 recent studies encompassing 1,731 DyeVert System cases and 1,387 control cases. Meta-analyses demonstrated that DyeVert System use: (a) reduced CMV delivered to the patient and CMV/baseline renal function ratios; (b) reduced the percentage of cases exceeding the maximum CMV threshold; and (c) maintained adequate image quality.

Additionally, DyeVert System use reduced CA-AKI incidence, resulting in a number needed to treat to avoid 1 CA-AKI event of 20. CA-AKI is associated with increased morbidity, mortality, and length of stay (6). Briguori et al. was the first report of a procedure-based CA-AKI prevention strategy resulting in significant CA-AKI reduction as well as a significantly shorter length of stay of about 2 days (30). Given the high cost of CA-AKI events (6), the overall economic value of the DyeVert System may be derived from CA-AKI avoidance based on recent hospital budget impact evaluations (32, 40, 41) and a modeling study (44).

Various intra-procedural contrast-sparing strategies have been suggested including limiting CMV per injection to 2 mL, use of optical coherence tomography, roadmap dynamic software, and biplane angiography (7). Additionally, use of automated CM injection systems may result in slightly less CMV over manual CM injection systems though the authors concluded it was unlikely to impact contrast-induced renal complications (45). Publications reviewed in this study demonstrate DyeVert System use as an additive strategy for reducing CMV and diversion of excess CMV is still significant even when other modalities are deployed.

Primary limitations of our review and meta-analysis include heterogenous definitions of CA-AKI, lack of reporting outcomes on potential subgroups of interest preclude further assessment of a potential treatment effect, small sample sizes of some studies, lack of long-term follow-up, clinical event committees not used, and lack of randomization in some studies. High *I*^2^ values (>75%), indicative of strong heterogeneity, were also seen in the meta-analyses of CMV threshold management in the intervention group, CMV/eGFR ratio, and in the analysis of CA-AKI incidence in the control group. However, given the small number of studies identified in the review and included in these primary analyses, further sub-group analyses of studies were not considered appropriate. Also, the scope of CA-AKI prevention strategies used in each case was not well reported in all studies and are often not specifically cited in the medical record, which precluded the ability to adjust for these potential variables. Additionally, we did not have access to patient-level data; and therefore, cannot confirm whether patients received optimal medical therapy for CA-AKI prevention. Despite these shortcomings, this exhaustive systematic review encompassing all relevant scientific databases identified numerous studies that demonstrated the clinical effectiveness of interventional use of the DyeVert System. Additionally, we used appropriate meta-analysis methods to synthesize outcomes of the included studies to thoroughly assess the efficacy and safety of the intervention.

In conclusion, DyeVert System use significantly reduces CMV delivered to the patient, CMV/baseline renal function ratios, and CA-AKI incidence while maintaining image quality. Accordingly, the device may serve as an adjunctive, procedure-based strategy to prevent CA-AKI. Future multi-center studies are needed to further assess effects of minimizing CMV on endpoints such as CA-AKI prevention, incidence of adverse cardiac and renal events, and health care costs.

## Data Availability Statement

The original contributions presented in the study are included in the article/Supplementary Material, further inquiries can be directed to the corresponding author/s.

## Author Contributions

AM, MJ, MY, and EM were responsible for performing the systematic review and meta-analysis. All other authors provided clinical input to the manuscript. All authors were responsible for developing the final, submitted version of the manuscript.

## Conflict of Interest

EM, AM, and MJ were employees of Optimax Access United Kingdom Ltd., and MY was employee of Optimax Access United Kingdom Ltd. and is now an Assistant Professor at Mercer University. AP, CB, and SB were paid consultants for Osprey Medical Corporation. AR and AP received honoraria from GE Healthcare as a consultant. RG has previously received speaker fees from Shockwave Medical Inc. Osprey Medical provided funding for the professional services of Optimax/Device Access, Wendy Mills Writing LLC, and Vita Medical LLC. The remaining authors declare that the research was conducted in the absence of any commercial or financial relationships that could be construed as a potential conflict of interest.

## Publisher’s Note

All claims expressed in this article are solely those of the authors and do not necessarily represent those of their affiliated organizations, or those of the publisher, the editors and the reviewers. Any product that may be evaluated in this article, or claim that may be made by its manufacturer, is not guaranteed or endorsed by the publisher.

## Abbreviations

CA: coronary angiography
CA-AKI: contrast-associated acute kidney injury
CM: contrast media
CMV: contrast media volume
eGFR: estimated glomerular filtration rate
PCI: percutaneous coronary intervention
PRISMA: Preferred Reporting Items for Systematic Reviews and Meta-Analyses
RCT: randomized controlled trial
RD: risk difference.
