# Supplementary material for: DyeVert Contrast Reduction System Use in Patients Undergoing Coronary and/or Peripheral Angiography: A Systematic Literature Review and Meta-Analysis
Source: Front Med (Lausanne). 2022 Apr 25;9:841876. doi: 10.3389/fmed.2022.841876 (PMC9081570; doi:10.3389/fmed.2022.841876)
Supplement: Supplementary file 1 [file Data_Sheet_1.docx]

# Supplemental Tables

Table S1. Search strategy in MEDLINE and MEDLINE In-Process (<1946 to July 15, 2021>)

| **Search #** | **Search terms** | **No. results** |
| --- | --- | --- |
| 1 | Acute Kidney Injury.mp. or Acute Kidney Injury/ | 60,735 |
| 2 | (contrast-induced* or radiocontrast-induced* or ci).mp. [mp=title, abstract, original title, name of substance word, subject heading word, floating sub-heading word, keyword heading word, organism supplementary concept word, protocol supplementary concept word, rare disease supplementary concept word, unique identifier, synonyms] | 1,615,958 |
| 3 | 1 and 2 | 5,803 |
| 4 | Contrast Media/ae | 10,079 |
| 5 | (ciaki or cin or ciraf or ci-aki or ci-arf or ci nephropathy* or cinephropath* or rci nephropathy* or rcinephropath*).mp. [mp=title, abstract, original title, name of substance word, subject heading word, floating sub-heading word, keyword heading word, organism supplementary concept word, protocol supplementary concept word, rare disease supplementary concept word, unique identifier, synonyms] | 28,518 |
| 6 | (aki or arf or acute kidney or acute renal or early kidney or early renal or necrosis or tubul*).mp. [mp=title, abstract, original title, name of substance word, subject heading word, floating sub-heading word, keyword heading word, organism supplementary concept word, protocol supplementary concept word, rare disease supplementary concept word, unique identifier, synonyms] | 1,405,691 |
| 7 | Acute Kidney Injury/ae | 0 |
| 8 | 3 or 4 or 5 or 6 or 7 | 1,437,218 |
| 9 | Dyevert.mp. | 22 |
| 10 | Osprey Medical.mp. | 268 |
| 11 | 9 or 10 | 278 |
| 12 | 8 and 11 | 55 |

Table S2. Search strategy in Embase (<1974 to July 15, 2021>)

| **Search #** | **Search terms** | **No. results** |
| --- | --- | --- |
| 1 | exp acute kidney failure/ | 102,418 |
| 2 | exp acute kidney tubule necrosis/ | 5,680 |
| 3 | 1 or 2 | 1,107,727 |
| 4 | (contrast-induced* or radiocontrast-induced* or ci).tw. | 922,760 |
| 5 | 3 and 4 | 11,916 |
| 6 | contrast medium/ae | 7,587 |
| 7 | contrast induced nephropathy/ | 6,172 |
| 8 | (ciaki or cin or ciraf or ci-aki or ci-arf or ci nephropath* or cinephropath* or rci nephropath* or rcinephropath*).tw. | 19,148 |
| 9 | (contrast-induced adj4 (aki or arf or acute kidney or acute renal or early kidney or early renal or necrosis or tubul*)).tw. | 1,615 |
| 10 | (radiocontrast-induced adj4 (aki or arf or acute kidney or acute renal or early kidney or early renal or necrosis or tubul*)).tw. | 75 |
| 11 | (radiocontrast* adj4 (nephropath* or nephrotoxi*)).tw. | 368 |
| 12 | 5 or 6 or 7 or 8 or 9 or 10 or 11 | 33,155 |
| 13 | Dyevert.mp. [mp=title, abstract, heading word, drug trade name, original title, device manufacturer, drug manufacturer, device trade name, keyword, floating subheading word, candidate term word] | 23 |
| 14 | 12 and 13 | 22 |

Table S3. Search strategy in Cochrane Library (CDSR and CENTRAL) (<to July 15, 2021>)

| **Search #** | **Search terms** | **No. results** |
| --- | --- | --- |
| 1 | Acute Kidney Injury.mp. [mp=ti, ot, ab, sh, hw, kw, tx, ct] | 4,559 |
| 2 | (contrast-induced* or radiocontrast-induced* or ci).mp. [mp=ti, ot, ab, sh, hw, kw, tx, ct] | 133,459 |
| 3 | 1 and 2 | 1,082 |
| 4 | Contrast Media.mp. [mp=ti, ot, ab, sh, hw, kw, tx, ct] | 6,965 |
| 5 | (ciaki or cin or ciraf or ci-aki or ci-arf or ci nephropath* or cinephropath* or rci nephropath* or rcinephropath*).mp. [mp=ti, ot, ab, sh, hw, kw, tx, ct] | 2,501 |
| 6 | radiocontrast-induced.mp. [mp=ti, ot, ab, sh, hw, kw, tx, ct] | 67 |
| 7 | (aki or arf or acute kidney or acute renal or early kidney or early renal or necrosis or tubul*).mp. [mp=ti, ot, ab, sh, hw, kw, tx, ct] | 39,855 |
| 8 | contrast*.mp. [mp=ti, ot, ab, sh, hw, kw, tx, ct] | 50,586 |
| 9 | (nephropath* or nephrotoxi*).mp. [mp=ti, ot, ab, sh, hw, kw, tx, ct] | 11,582 |
| 10 | radiocontrast*.mp. [mp=ti, ot, ab, sh, hw, kw, tx, ct] | 255 |
| 11 | (nephropath* or nephrotoxi*).mp. [mp=ti, ot, ab, sh, hw, kw, tx, ct] | 11,582 |
| 12 | 3 or 4 or 5 or 6 or 7 or 8 or 9 or 10 or 11 | 87,098 |
| 13 | Dyevert.mp. [mp=ti, ot, ab, sh, hw, kw, tx, ct] | 8 |
| 14 | 12 and 13 | 7 |

Table S4. Search strategy in ClinicalTrials.gov (<to July 15, 2021>)

| **Search #** | **Search terms** | **No. results** |
| --- | --- | --- |
| 1 | Dyevert | 6 |

Table S5. Search strategy in International Clinical Trials Registry Platform (<to July 15, 2021>)

| **Search #** | **Search terms** | **No. results** |
| --- | --- | --- |
| 1 | Dyevert | 7 |

Table S6. Additional baseline and procedure characteristics

| **Study author (year)** | **Study arm** | **Dialysis (%)** | **Anemia (%)** | **PAD (%)** | **Prior CABG (%)** | **Prior MI (%)** | **Total fluoroscopy time (min)** | **STEMI (%)** | **Hemo-dynamic support (%)** |  |  |  |  |  |  |  |
| --- | --- | --- | --- | --- | --- | --- | --- | --- | --- | --- | --- | --- | --- | --- | --- | --- |
| Desch (2018) | DyeVert | NR | 10 | 17 | 0 | NR | 3.9 ± 3.9 | 0 | 0 |  |  |  |  |  |  |  |
|  | Control | NR | 17 | 17 | 0 | NR | 3.7 ± 3.5 | 0 | 0 |  |  |  |  |  |  |  |
| Bath (2019) | DyeVert | NR | NR | NR | NR | NR | NR | NR | NR |  |  |  |  |  |  |  |
|  | Control | NR | NR | NR | NR | NR | NR | NR | NR |  |  |  |  |  |  |  |
| Briguori (2020) | DyeVert | NR | NR | 21 | NR | NR | NR | 85 | 6 |  |  |  |  |  |  |  |
|  | Control | NR | NR | 20 | NR | NR | NR | 81 | 4 |  |  |  |  |  |  |  |
| Sattar (2018) | DyeVert | NR | NR | NR | NR | NR | NR | NR | NR |  |  |  |  |  |  |  |
|  | Control | NR | NR | NR | NR | NR | NR | NR | NR |  |  |  |  |  |  |  |
| Kutschman (2019) | Overall | NR | NR | NR | NR | NR | NR | NR | NR |  |  |  |  |  |  |  |
| Kutschman (2019) | Overall | 9 | 7 | 5 | 23 | NR | NR | 10 | 4 |  |  |  |  |  |  |  |
| Bunney (2019) | DyeVert | NR | NR | NR | NR | NR | NR | NR | 20 |  |  |  |  |  |  |  |
|  | Control | NR | NR | NR | NR | NR | NR | NR | 4 |  |  |  |  |  |  |  |
| Tajti (2019) | DyeVert | 3 | NR | 21 | 49 | 54 | 59.6 (27.0-90.4) | NR | NR |  |  |  |  |  |  |  |
|  | Control | 3 | NR | 6 | 37 | 34 | 41.6 (28.0-73.5) | NR | NR |  |  |  |  |  |  |  |
| Zimin (2020) | DyeVert | 7 | NR | 7 | 7 | NR | NR | NR | NR |  |  |  |  |  |  |  |
| Sapontis (2017) | DyeVert | 2 | NR | 9 | 9 | 30 | 8.6 ± 7.2 | 0 | NR |  |  |  |  |  |  |  |
| Corcione (2017) | DyeVert | NR | NR | NR | NR | NR | NR | NR | NR |  |  |  |  |  |  |  |
| Gurm (2019) | DyeVert | 0 | 29 | NR | 35 | 34 | 12.8 ± 14.4 | 0 | NR |  |  |  |  |  |  |  |
| Turner/Tucker (2020) | Overall | NR | NR | NR | NR | NR | NR | NR | NR |  |  |  |  |  |  |  |
| Cameron (2020) | Overall | NR | NR | NR | NR | NR | NR | NR | NR |  |  |  |  |  |  |  |
| Rao (2019) | DyeVert | NR | NR | 71 | 29 | 29 | NR | 0 | 0 |  |  |  |  |  |  |  |
| Amoroso (2020) | DyeVert | NR | NR | NR | NR | NR | NR | 0 | 0 |  |  |  |  |  |  |  |
| Bruno (2019) | DyeVert | NR | NR | NR | NR | NR | NR | 0 | 0 |  |  |  |  |  |  |  |

Data presented as mean ± standard deviation, median (IQR) or %.

Abbreviations: CABG, coronary artery bypass graft; MI, myocardial infarction; NR, not reported; PAD, peripheral arterial disease; STEMI, ST-elevation myocardial infarction.

Table S7. Contrast-associated acute kidney injury prevention strategies for studies reporting contrast-associated acute kidney injury rates

| **Study author (year)** | **Risk screening** | **Contrast media threshold** | **Hydration** |
| --- | --- | --- | --- |
| Briguori (2020) | ACS patients (STEMI/NSTEMI)  with Mehran score ≥11 and Gurm score ≥7 | < 3x eGFR | 0.9% sodium chloride, 3 mL/kg/h (or ≤ 1.5 mL/kg/h if LVEF <40%) prior, intra-procedural rate was further adjusted according to LVEDP as follows: 5 ml/kg/h for LVEDP ≤12 mmHg, irrespective to LVEF; 3 ml/kg/h for LVEDP 13–18 mmHg and/or LVEF <40%; and 1.5 ml/kg/h for LVEDP >18 mmHg, irrespective to the LVEF value. Target >960 mL/case. |
|  |  |  |  |
|  |  |  |  |
| Sattar (2018) | PCI cases with eGFR <60 | NR | Per treating physician |
| Kutschman (2019) | Inpatients and outpatients with CKD, renal transplant, complex multivessel disease, STEMI/ NSTEMI, or recent contrast study | <3x eGFR | 0.9% sodium chloride,  1 mL/kg/h in non-CHF or 0.5 mL/kg/h in CHF for 12 h prior; post per treating physician |
| Bunney (2019) | NR | NR | NR |
|  |  |  |  |
| Tajti (2019) | NR | NR | NR |
|  |  |  |  |
| Corcione (2017) | NR | NR | NR |
|  |  |  |  |
| Gurm (2019) | eGFR 20-60 | <3x eGFR,  <3.7x eGFR, or physician discretion | Per treating physician |
|  |  |  |  |
| Turner/Tucker (2020) | eGFR <60, SCr >1.5, or STEMI, use of SCAI risk prediction tool | <3x eGFR | 3 mg/kg 1 h prior; intra and 4 h post LVEDP-based |
| Cameron (2020) | eGFR <60 | <3x eGFR | Per treating physician |
| Rao (2019) | Complex PVI | NR | NR |

Abbreviations: ACS, acute coronary syndrome; CHF, congestive heart failure; CKD, chronic kidney disease; eGFR, estimated glomerular filtration rate; LVEDP, left ventricular end diastolic pressure; LVEF, left ventricular ejection fraction; NR, not reported; NSTEMI, non ST-elevation myocardial infarction; PCI, percutaneous coronary intervention; PVI, peripheral vascular intervention; SCAI, Society for Cardiovascular Angiography and Interventions; SCr, serum creatinine; STEMI, ST-elevation myocardial infarction

Table S8. Results: Contrast media volume/baseline renal function ratio

| **Study/author (year)** | **Study arm** | **Actual CMV/ eGFR (mean)** | **Attempted CMV/ eGFR (mean)** | **Actual CMV/ eGFR ≤3 (%)** | **Attempted CMV/ eGFR ≤3 (%)** | **Actual CMV/ eGFR ≤2 (%)** | **Attempted CMV/ eGFR ≤2 (%)** | **Actual CMV/ eGFR ≤1 (%)** | **Attempted CMV/ eGFR ≤1 (%)** |
| --- | --- | --- | --- | --- | --- | --- | --- | --- | --- |
| Desch (2018) | DyeVert | NR | NR | NR | NR | NR | NR | NR | NR |
|  | Control | NR | NA | NR | NA | NR | NA | NR | NA |
|  |  |  |  |  |  |  |  |  |  |
| Bath (2019) | DyeVert | NR | NR | NR | NR | NR | NR | NR | NR |
|  | Control | NR | NA | NR | NA | NR | NA | NR | NA |
|  |  |  |  |  |  |  |  |  |  |
| Briguori (2020) | DyeVert | NR | NR | 53 | NR | NR | NR | NR | NR |
|  | Control | NR | NA | 40 | NA | NR | NA | NR | NA |
|  |  |  |  |  |  |  |  |  |  |
| Sattar (2018) | DyeVert | NR | NR | NR | NR | NR | NR | NR | NR |
|  | Control | NR | NA | NR | NA | NR | NA | NR | NA |
|  |  |  |  |  |  |  |  |  |  |
| Kutschman (2019) | DyeVert | 2.5 ± 1.8 | 3.7 ± 3.0 | NR | NR | NR | NR | NR | NR |
|  | Control | 3.7 ± 5.3 | NA | NR | NA | NR | NA | NR | NA |
|  |  |  |  |  |  |  |  |  |  |
| Kutschman (2019) | DyeVert | 2.1 ± 2.2 | 3.1 ± 3.2 | 83 | 64 | 60 | 43 | 30 | 19 |
|  | Control | 3.2 ± 5.6 | NA | NR | NA | NR | NA | NR | NA |
|  |  |  |  |  |  |  |  |  |  |
| Bunney (2019) | DyeVert | NR | NR | NR | NR | NR | NR | NR | NR |
|  | Control | NR | NA | NR | NA | NA | NA | NA | NA |
|  |  |  |  |  |  |  |  |  |  |
| Tajti (2019) | DyeVert | NR | NR | NR | NR | NR | NR | NR | NR |
|  | Control | NR | NA | NR | NA | NA | NA | NA | NA |
|  |  |  |  |  |  |  |  |  |  |
| Zimin (2020) | DyeVert | 3.1 ± 1.3 | 5.0 ± 1.9 | 69 | 6 | 0 | 0 | 0 | 0 |
|  |  |  |  |  |  |  |  |  |  |
| Sapontis (2017) | DyeVert | NR | NR | NR | NR | NR | NR | NR | NR |
|  |  |  |  |  |  |  |  |  |  |
| Corcione (2017) | DyeVert | 1.0 ± 0.6 | 1.8 ± 0.9 | 90 | 90 | 90 | 60 | 60 | 40 |
|  |  |  |  |  |  |  |  |  |  |
| Gurm (2019) | DyeVert | 1.6 ± 1.1 | 2.7 ± 1.7 | 91 | 74 | 75 | 41 | 33 | 7 |
|  |  |  |  |  |  |  |  |  |  |
| Turner/Tucker (2020) | DyeVert | 2.1 ± 1.5 | 3.1 ± 2.0 | 82 | 62 | 60 | 33 | 18 | 6 |
|  |  |  |  |  |  |  |  |  |  |
| Cameron (2020) | DyeVert | 2.1 ± 2.1 | 3.4 ± 3.0 | 84 | 53 | 61 | 35 | 27 | 3 |
|  |  |  |  |  |  |  |  |  |  |
| Rao (2019) | DyeVert | NR | NR | NR | NR | NR | NR | NR | NR |
|  |  |  |  |  |  |  |  |  |  |
| Amoroso (2020) | DyeVert | NR | NR | NR | NR | NR | NR | NR | NR |
|  |  |  |  |  |  |  |  |  |  |
| Bruno (2019) | DyeVert | 1.1 | 1.8 | NR | NR | NR | NR | NR | NR |

Data presented are mean, mean ± standard deviation, or %.

Abbreviations: CMV, contrast media volume; eGFR, estimated glomerular filtration rate; NA, not applicable; NR, not reported.

**Table S9.** Results: Image quality

|  |  | **Adequate image quality per**  **physician assessment** | | **Adequate image quality per independent reviewer** | |
| --- | --- | --- | --- | --- | --- |
| **Study author (year)** | **Study arm** | **(% of cases)** | **(% of images)** | **(% of cases)** | **(% of images)** |
| Desch (2018) | DyeVert | NR | 96 | NR | 90.7 |
|  | Control | NR | 95 | NR | 97.3 |
|  |  |  |  |  |  |
| Briguori (2020) | DyeVert | 100 | NA | NA | NA |
|  | Control | NR | NA | NA | NA |
|  |  |  |  |  |  |
| Zimin (2020) | DyeVert | 100 | NA | NA | CIL:86.6 ± 15.6  CSL: 92.1 ± 14.8  CROI: 95.7 ± 9.9 |
|  | Control | NR | NA | NA | CIL: 90.9 ± 10.4  CSL: 95.3 ± 11.3  CROI: 97.7 ± 7.6 |
|  |  |  |  |  |  |
| Sapontis (2017) | DyeVert | 98 | NA | NA | NA |
|  |  |  |  |  |  |
| Corcione (2017) | DyeVert | 100 | NA | NA | NA |
|  |  |  |  |  |  |
| Gurm (2019) | DyeVert | 99 | NA | NA | NA |
|  |  |  |  |  |  |
| Rao (2019) | DyeVert | 100 | NA | NA | NA |
|  |  |  |  |  |  |
| Amoroso (2020) | DyeVert | 96 | NA | NA | NA |
|  |  |  |  |  |  |
| Bruno (2019) | DyeVert | 100 | NA | NA | NA |

Data presented are mean ± standard deviation or %.

Abbreviations: CIL, clear image length; CROI, clear region of interest; CSL, clear stent length; NA, not applicable; NR, not reported.

**Table S10.** Results: Contrast-associated acute kidney injury definition and rates

| **Study author (year)** | **CA-AKI**  **definition** | **Study arm** | **Rate (%)** |
| --- | --- | --- | --- |
| Briguori (2020) | SCr ↑ ≥ 0.3 mg/dL within 72 hours | DyeVert | 8 |
|  |  | Control | 19 |
| Sattar (2018) | SCr ↑ ≥ 0.3 mg/dL or 50% | DyeVert | 12 |
|  |  | Control | 16 |
| Kutschman (2019) | SCr ↑ ≥ 0.3 mg/dL or 50% within 48 hours | DyeVert | 9 |
|  |  | Control | 22 |
| Kutschman (2019) | SCr ↑ ≥ 0.3 mg/dL or 50% within 48 hours | DyeVert | 7 |
|  |  | Control | 10 |
| Bunney (2019) | SCr ↑ ≥ 0.3 mg/dL or 50% within 48 hours | DyeVert | 3 |
|  |  | Control | 9 |
| Tajti (2019) | NR | DyeVert | In-Hospital: 1,  Post-Procedure: 3 |
|  |  | Control | In-Hospital: 2,  Post-Procedure: 2 |
| Corcione (2017) | NR | DyeVert | 10 |
| Gurm (2019) | SCr ↑ ≥ 0.5 mg/dL through discharge | DyeVert | 11 |
| Turner (2020) | SCr ↑ ≥ 0.3 mg/dL or 50% within 48 hours | Overall | Initial: 13  Final: 2 |
| Cameron (2020) | SCr ↑ ≥ 0.3 mg/dL or 50% within 48 hours | Overall | Initial: 11  Final: 5 |
| Rao (2019) | Worsening renal function | DyeVert | 0 |

Abbreviations: CA-AKI, contrast-associated acute kidney injury; NR, not reported; SCr, serum creatinine.

Table S11. Meta-analysis: Mean difference in absolute contrast volume (ml) in randomized controlled trials

| Study | N1 | N2 | Total | SMD | SE | 95% CI | t | P | Weight (%) | |
| --- | --- | --- | --- | --- | --- | --- | --- | --- | --- | --- |
|  |  |  |  |  |  |  |  |  | **Fixed** | **Random** |
| Desch (2018) | 48 | 48 | 96 | -2.146 | 0.255 | -2.652 to -1.640 |  |  | 49.33 | 49.33 |
| Bath (2019) | 49 | 59 | 108 | -2.389 | 0.252 | -2.888 to -1.891 |  |  | 50.67 | 50.67 |
| Total (fixed effects) | 97 | 107 | 204 | -2.269 | 0.179 | -2.622 to -1.916 | -12.674 | <0.001 | 100.00 | 100.00 |
| Total (random effects) | 97 | 107 | 204 | -2.269 | 0.179 | -2.622 to -1.916 | -12.674 | <0.001 | 100.00 | 100.00 |

Abbreviations: CI, confidence interval; N1, DyeVert Group sample size; N2, Control Group sample size; SMD, standardized mean difference; SE, standard error.

**Test for heterogeneity**

| Q | 0.4618 |
| --- | --- |
| DF | 1 |
| Significance level | P = 0.4968 |
| I^2^ (inconsistency) | 0.00% |
| 95% CI for I^2^ | 0.00 to 0.00 |

**Publication bias**

| Egger's test | |
| --- | --- |
| Intercept | 71.2977 |
| 95% CI |  |
| Significance level | P < 0.0001 |
| Begg’s test | |
| Kendall's Tau | 1.0000 |
| Significance level | P = 0.3173 |

Table S12. Meta-analysis: Mean difference in absolute contrast media volume (ml) in observational, two-arm studies

| Study | N1 | N2 | Total | SMD | SE | 95% CI | t | P | Weight (%) | |
| --- | --- | --- | --- | --- | --- | --- | --- | --- | --- | --- |
|  |  |  |  |  |  |  |  |  | **Fixed** | **Random** |
| Briguori (2020) | 90 | 90 | 180 | -0.617 | 0.152 | -0.917 to -0.317 |  |  | 22.40 | 32.13 |
| Tajti (2019) | 39 | 91 | 130 | -0.769 | 0.196 | -0.1157 to -0.0381 |  |  | 13.45 | 25.91 |
| Kutschman (2019) | 258 | 243 | 501 | -0.315 | 0.089 | -0.491 to -0.138 |  |  | 64.15 | 41.97 |
| Total (fixed effects) | 387 | 424 | 811 | -0.443 | 0.071 | -0.585 to -0.302 | -6.165 | <0.001 | 100.00 | 100.00 |
| Total (random effects) | 387 | 424 | 811 | -0.529 | 0.143 | -0.811 to -0.248 | -3.690 | <0.001 | 100.00 | 100.00 |

Abbreviations: CI, confidence interval; N1, DyeVert Group sample size; N2, Control Group sample size; SMD, standardized mean difference; SE, standard error.

**Test for heterogeneity**

| Q | 6.1204 |
| --- | --- |
| DF | 2 |
| Significance level | P = 0.0496 |
| I^2^ (inconsistency) | 67.32% |
| 95% CI for I^2^ | 0.00 to 90.55 |

**Publication bias**

| Egger's test | |
| --- | --- |
| Intercept | -4.4499 |
| 95% CI | -8.8678 to -0.03205 |
| Significance level | P =0.0496 |
| Begg’s test | |
| Kendall's Tau | -1.0000 |
| Significance level | P = 0.1172 |

**Table S13.** Meta-analysis: DyeVert group Proportion of attempted contrast media volume diverted by the DyeVert System (%) in observational studies

A) DyeVert Products Used with Manual and Automated CM Injection Systems

| Study | Sample size | Proportion of attempted CMV diverted (%) | 95% CI | Weight (%) | |
| --- | --- | --- | --- | --- | --- |
|  |  |  |  | **Fixed** | **Random** |
| Sapontis (2017) | 44 | 47.700 | 32.436 to 63.285 | 5.17 | 5.17 |
| Corcione (2017) | 10 | 41.800 | 13.260 to 75.189 | 1.26 | 1.26 |
| Gurm (2019) | 114 | 40.100 | 31.032 to 49.695 | 13.20 | 13.20 |
| Cameron (2020) | 423 | 38.000 | 33.354 to 42.815 | 48.68 | 48.68 |
| Kutschman (2019) | 258 | 40.000 | 33.974 to 46.258 | 29.74 | 29.74 |
| Zimin (2020) | 16 | 37.500 | 15.198 to 64.565 | 1.95 | 1.95 |
| Amoroso (2020) | 26 | 34.40 | 17.038 to 55.438 | 3.01 | 3.01 |
| Total (fixed effects) | 891 | 39.266 | 36.056 to 42.547 | 100.00 | 100.00 |
| Total (random effects) | 891 | 39.266 | 36.097 to 42.481 | 100.00 | 100.00 |

Abbreviations: CI, confidence interval; CMV, contrast media volume.

**Test for heterogeneity**

| Q | 1.9355 |
| --- | --- |
| DF | 6 |
| Significance level | P = 0.9255 |
| I^2^ (inconsistency) | 0.00% |
| 95% CI for I^2^ | 0.00 to 11.01 |

| Egger’s test | |
| --- | --- |
| Intercept | 0.3147 |
| 95% CI | -0.7457 to 1.3750 |
| Significance level | P = 0.4800 |
| Begg’s test | |
| Kendall’s Tau | 0.1429 |
| Significance level | P = 0.6523 |

**Publication bias**

B) DyeVert Products Used with Manual CM Injection Systems

| Study | Sample size | Proportion of attempted CMV diverted (%) | 95% CI | Weight (%) | |
| --- | --- | --- | --- | --- | --- |
|  |  |  |  | **Fixed** | **Random** |
| Sapontis (2017) | 44 | 47.700 | 32.436 to 63.285 | 5.17 | 5.17 |
| Corcione (2017) | 10 | 41.800 | 13.260 to 75.189 | 1.26 | 1.26 |
| Gurm (2019) | 114 | 40.100 | 31.032 to 49.695 | 13.20 | 13.20 |
| Cameron (2020) | 423 | 38.000 | 33.354 to 42.815 | 48.68 | 48.68 |
| Kutschman (2019) | 258 | 40.000 | 33.974 to 46.258 | 29.74 | 29.74 |
| Zimin (2020) | 16 | 37.500 | 15.198 to 64.565 | 1.95 | 1.95 |
| Total (fixed effects) | 865 | 39.465 | 36.202 to 42.799 | 100.00 | 100.00 |
| Total (random effects) | 865 | 39.465 | 36.244 to 42.732 | 100.00 | 100.00 |

Abbreviations: CI, confidence interval; CMV, contrast media volume.

**Test for heterogeneity**

| Q | 1.7421 |
| --- | --- |
| DF | 5 |
| Significance level | P = 0.8836 |
| I^2^ (inconsistency) | 0.00% |
| 95% CI for I^2^ | 0.00 to 29.27 |

| Egger’s test | |
| --- | --- |
| Intercept | 0.5828 |
| 95% CI | -0.5922 to 1.7578 |
| Significance level | P = 0.2405 |
| Begg’s test | |
| Kendall’s Tau | 0.2000 |
| Significance level | P = 0.5730 |

**Publication bias**

**Table S14.** Meta-analysis: Image quality in the DyeVert group (Proportion of cases with adequate image quality per physician assessment)

| Study | Sample size | Proportion of cases with adequate image quality (%) | 95% CI | Weight (%) | |
| --- | --- | --- | --- | --- | --- |
|  |  |  |  | **Fixed** | **Random** |
| Desch (2018) | 48 | 95.500 | 85.262 to 99.382 | 13.80 | 13.94 |
| Briguori (2020) | 90 | 100.000 | 95.984 to 100.000 | 25.63 | 25.49 |
| Sapontis (2017) | 44 | 97.727 | 87.976 to 99.942 | 12.68 | 12.82 |
| Corcione (2017) | 10 | 100.000 | 69.150 to 100.000 | 3.10 | 3.17 |
| Gurm (2019) | 114 | 99.123 | 95.209 to 99.978 | 32.39 | 31.93 |
| Zimin (2020) | 16 | 100.000 | 79.409 to 100.000 | 4.79 | 4.90 |
| Amoroso (2020) | 26 | 96.154 | 80.363 to 99.903 | 7.61 | 7.75 |
| Total (fixed effects) | 348 | 98.222 | 96.239 to 99.324 | 100.00 | 100.00 |
| Total (random effects) | 348 | 98.207 | 96.542 to 99.337 | 100.00 | 100.00 |

Abbreviations: CI, confidence interval.

**Test for heterogeneity**

| Q | 6.1062 |
| --- | --- |
| DF | 6 |
| Significance level | P = 0.4114 |
| I^2^ (inconsistency) | 1.74% |
| 95% CI for I^2^ | 0.00 to 71.79 |

**Publication bias**

| Egger’s test |  |
| --- | --- |
| Intercept | -1.2793 |
| 95% CI | -3.9244 to 1.3659 |
| Significance level | P = 0.2689 |
| Begg’s test |  |
| Kendall’s Tau | -0.1429 |
| Significance level | P = 0.6523 |

**Table S15.** Meta-analysis: Pooled estimate of contrast-associated acute kidney injury incidence in DyeVert group

a) Five Studies, Primary Analysis

| Study | Sample size | Proportion of cases with  CA-AKI (%) | 95% CI | Weight (%) | |
| --- | --- | --- | --- | --- | --- |
|  |  |  |  | **Fixed** | **Random** |
| Briguori (2020) | 90 | 8.000 | 3.328 to 15.652 | 19.70 | 19.70 |
| Kutschman (2019) | 258 | 6.899 | 4.126 to 10.710 | 55.06 | 56.06 |
| Sattar (2018) | 41 | 12.195 | 4.081 to 26.204 | 9.09 | 9.09 |
| Bunney (2019) | 29 | 3.448 | 0.0873 to 17.764 | 6.49 | 6.49 |
| Tajti (2019) | 39 | 2.564 | 0.0649 to 13.476 | 8.66 | 8.66 |
| Total (fixed effects) | 457 | 7.302 | 5.102 to 10.067 | 100.00 | 100.00 |
| Total (random effects) | 457 | 7.302 | 5.111 to 9.849 | 100.00 | 100.00 |

Abbreviations: CA-AKI, contrast-associated acute kidney injury; CI, confidence interval.

**Test for heterogeneity**

| Q | 3.1240 |
| --- | --- |
| DF | 4 |
| Significance level | P = 0.5373 |
| I^2^ (inconsistency) | 0.00% |
| 95% CI for I^2^ | 0.00 to 74.93 |

**Publication bias**

| Egger’s test |  |
| --- | --- |
| Intercept | -0.1021 |
| 95% CI | -3.6713 to 3.4671 |
| Significance level | P = 0.9332 |
| Begg’s test |  |
| Kendall’s Tau | -0.2000 |
| Significance level | P = 0.6242 |

B) Four Studies, Excluding Tajti (2019)

| Study | Sample size | Proportion of cases with  CA-AKI (%) | 95% CI | Weight (%) | |
| --- | --- | --- | --- | --- | --- |
|  |  |  |  | **Fixed** | **Random** |
| Briguori (2020) | 90 | 8.000 | 3.328 to 15.652 | 21.56 | 21.56 |
| Kutschman (2019) | 258 | 6.900 | 4.127 to 10.711 | 61.37 | 61.37 |
| Sattar (2018) | 41 | 12.200 | 4.084 to 26.211 | 9.95 | 9.95 |
| Bunney (2019) | 29 | 3.450 | 0.0874 to 17.767 | 7.11 | 7.11 |
| Total (fixed effects) | 418 | 7.710 | 5.351 to 10.682 | 100.00 | 100.00 |
| Total (random effects) | 418 | 7.710 | 5.361 to 10.444 | 100.00 | 100.00 |

Abbreviations: CA-AKI, contrast-associated acute kidney injury; CI, confidence interval.

**Test for heterogeneity**

| Q | 1.9621 |
| --- | --- |
| DF | 3 |
| Significance level | P = 0.5803 |
| I^2^ (inconsistency) | 0.00% |
| 95% CI for I^2^ | 0.00 to 80.26 |

**Publication bias**

| Egger’s test |  |
| --- | --- |
| Intercept | 0.5620 |
| 95% CI | -4.4335 to 5.5574 |
| Significance level | P = 0.06762 |
| Begg’s test |  |
| Kendall’s Tau | 0.0000 |
| Significance level | P = 1.0000 |

Table S16. Meta-analysis: Pooled estimate of contrast-associated acute kidney injury incidence in Control group

A) Five Studies, Primary Analysis

| Study | Sample size | Proportion of cases with  CA-AKI (%) | 95% CI | Weight (%) | |
| --- | --- | --- | --- | --- | --- |
|  |  |  |  | **Fixed** | **Random** |
| Briguori (2020) | 90 | 19.000 | 11.495 to 28.636 | 7.18 | 17.71 |
| Kutschman (2019) | 243 | 10.300 | 6.779 to 14.826 | 19.26 | 22.79 |
| Sattar (2018) | 68 | 16.206 | 8.384 to 27.137 | 5.45 | 15.91 |
| Bunney (2019) | 770 | 9.351 | 7.388 to 11.631 | 60.85 | 25.80 |
| Tajti (2019) | 91 | 2.198 | 0.267 to 7.715 | 7.26 | 17.78 |
| Total (fixed effects) | 1262 | 9.936 | 8.343 to 11.716 | 100.00 | 100.00 |
| Total (random effects) | 1262 | 10.648 | 6.602 to 15.521 | 100.00 | 100.00 |

Abbreviations: CA-AKI, contrast-associated acute kidney injury; CI, confidence interval.

**Test for heterogeneity**

| Q | 18.6356 |
| --- | --- |
| DF | 4 |
| Significance level | P = 0.0009 |
| I^2^ (inconsistency) | 78.54% |
| 95% CI for I^2^ | 48.67 to 91.02 |

**Publication bias**

| Egger’s test |  |
| --- | --- |
| Intercept | 0.9740 |
| 95% CI | -6.5916 to 8.5397 |
| Significance level | P = 0.7095 |
| Begg’s test |  |
| Kendall’s Tau | 0.4000 |
| Significance level | P = 0.3272 |

B) Four Studies, Excluding Tajti (2019)

| Study | Sample size | Proportion of cases with  CA-AKI (%) | 95% CI | Weight (%) | |
| --- | --- | --- | --- | --- | --- |
|  |  |  |  | **Fixed** | **Random** |
| Briguori (2020) | 90 | 19.000 | 11.495 to 28.636 | 7.74 | 19.16 |
| Kutschman (2019) | 243 | 10.300 | 6.779 to 14.826 | 20.77 | 28.62 |
| Sattar (2018) | 68 | 16.200 | 8.379 to 27.131 | 5.87 | 16.41 |
| Bunney (2019) | 770 | 9.350 | 7.388 to 11.630 | 65.62 | 35.81 |
| Total (fixed effects) | 1171 | 10.677 | 8.969 to 12.585 | 100.00 | 100.00 |
| Total (random effects) | 1171 | 12.552 | 8.745 to 16.934 | 100.00 | 100.00 |

Abbreviations: CA-AKI, contrast-associated acute kidney injury; CI, confidence interval.

**Test for heterogeneity**

| Q | 8.9892 |
| --- | --- |
| DF | 3 |
| Significance level | P = 0.0294 |
| I^2^ (inconsistency) | 66.63% |
| 95% CI for I^2^ | 2.38 to 88.59 |

**Publication bias**

| Egger’s test |  |
| --- | --- |
| Intercept | 3.1018 |
| 95% CI | -0.6136 to 6.8172 |
| Significance level | P = 0.0695 |
| Begg’s test |  |
| Kendall’s Tau | 0.6667 |
| Significance level | P = 0.1742 |

Table S17. Meta-analysis: Pooled estimate of the absolute risk reduction and relative risk of contrast-associated acute kidney injury in DyeVert group versus control group

A) Five Studies, Primary Analysis

| **Study author (Year)** |  | | **CA-AKI Absolute risk reduction (%)  (95% CI)** | **Relative risk of a CA-AKI event  (95% CI)** | **NNT to avoid 1 CA-AKI event** |
| --- | --- | --- | --- | --- | --- |
|  | **Proportion of Cases  with CA-AKI (%)** | |  |  |  |
|  | **DyeVert Group**  **(95% CI)** | **Control Group**  **(95% CI)** |  |  |  |
| Briguori (2020) | 8.000 (3.328 - 15.652) | 19.000  (11.495 - 28.636) | 11.00  (-3.73 to 25.73) | 0.42  (0.18-0.95) | 9 |
|  |  |  |  |  |  |
| Kutschman (2019) | 6.899  (4.126 - 10.710) | 10.300  (6.779 - 14.826) | 3.00  (-5.78 to 11.78) | 0.67  (0.37-1.20) | 29 |
|  |  |  |  |  |  |
| Sattar (2018) | 12.195 (4.081 - 26.204) | 16.206  (8.384 - 27.137) | 4.00  (-15.04 to 23.04) | 0.75  (0.28-2.01) | 25 |
|  |  |  |  |  |  |
| Bunney (2019) | 3.448 (0.0873 - 17.764) | 9.351  (7.388 - 11.631) | 6.00  (-0.95 to 12.95) | 0.37  (0.05-2.56) | 17 |
|  |  |  |  |  |  |
| Tajti (2019) | 2.564  (0.0649 - 13.476) | 2.198  (0.267 - 7.715) | -0.4.00  (-2.00 to 2.20) | 1.17  (0.11-12.5) | -273 |
|  |  |  |  |  |  |
| Pooled | 7.302 (5.111 - 9.849) | 10.648  (6.602 - 15.521) | 5.00  (0.40 to 9.80) | 0.60  (0.40-0.90) | 20 |

| **Absolute risk reduction** | **Fisher Z** | **r** | **SE** | **95% CI** | **z score** | **p value** | **Heterogeneity** |
| --- | --- | --- | --- | --- | --- | --- | --- |
| Fixed Effect Model | 0.05 | 0.05 | 0.024 | [0.004,0.098] | 2.113 | 0.0346 | I^2^=0.0%, Chi^2^=0.0, df=4 |
| Random Effect Model | 0.05 | 0.05 | 0.024 | [0.004,0.098] | 2.113 | 0.0346 | I^2^=0.0%, Tau^2^=0.0 |
| **Relative risk** | **RR** | **P** |  | **95% CI** | **z score** | **p value** | **Heterogeneity** |
| Fixed Effect Model | 0.59 | 0.01 |  | [0.39,0.89] | -2.50 | 0.08 | I^2^=0.0%, Chi^2^=0.0, df=3 |
| Random Effect Model | 0.60 | 0.01 |  | [0.40,0.90] | -2.41 | 0.08 | I^2^=0.0%, Tau^2^=0.0 |

Abbreviations: CA-AKI, contrast-associated acute kidney injury; CI, confidence interval; DF, degrees of freedom; NNT, number needed-to-treat; NR, not reported; RR, relative risk; SCr, serum creatinine; SE, standard error.

B) Four Studies, Excluding Tajti (2019)

|  | **Proportion of Cases  with CA-AKI (%)** | | | **CA-AKI Absolute risk reduction (%) (95% CI)** | | | **Relative risk of a CA-AKI event  (95% CI)** | | **NNT to avoid 1  CA-AKI event** | |  |
| --- | --- | --- | --- | --- | --- | --- | --- | --- | --- | --- | --- |
| **Studies** | **DyeVert Group**  **(95% CI)** | **Control Group**  **(95% CI)** | |  |  |  |  |  |  |  |  |
| Briguori (2020) | 8.000 (3.328 - 15.652) | 19.000  (11.495 - 28.636) | | 11.00  (-3.73 to 25.73) | | | 0.42  (0.18-0.95) | | 9 | |  |
|  |  |  | |  | | |  | |  | |  |
| Kutschman (2019) | 6.899  (4.126 - 10.710) | 10.300  (6.779 - 14.826) | | 3.00  (-5.78 to 11.78) | | | 0.67  (0.37-1.20) | | 29 | |  |
|  |  |  | |  | | |  | |  | |  |
| Sattar (2018) | 12.195 (4.081 - 26.204) | 16.206  (8.384 - 27.137) | | 4.00  (-15.04 to 23.04) | | | 0.75  (0.28-2.01) | | 25 | |  |
|  |  |  | |  | | |  | |  | |  |
| Bunney (2019) | 3.448 (0.0873 - 17.764) | 9.351  (7.388 - 11.631) | | 6.00  (-0.95 to 12.95) | | | 0.37  (0.05-2.56) | | 17 | |  |
|  |  |  | |  | | |  | |  | |  |
| Pooled | 7.710  (5.361 – 10.444) | 12.552  (8.745 – 16.934) | | 5.00 (0.60 to 10.40) | | | 0.59  (0.38-0.89) | | 20 | |  |
| **Absolute risk reduction** | **Fisher Z** | **r** | **SE** | | **95% CI** | **z score** | | **p value** | | **Heterogeneity** | |
| Fixed Effect Model | 0.05 | 0.05 | 0.025 | | [0.006,0.104] | 2.18 | | 0.0293 | | I^2^=0.0%, Chi^2^=0.0, df=3 | |
| Random Effect Model | 0.05 | 0.05 | 0.025 | | [0.006,0.104] | 2.18 | | 0.0293 | | I^2^=0.0%, Tau^2^=0.0 | |
| **Relative risk** | **RR** | **P** |  | | **95% CI** | **z score** | | **p value** | | **Heterogeneity** | |
| Fixed Effect Model | 0.57 | 0.01 |  | | [0.38,0.87] | -2.56 | | 0.072 | | I^2^=0.0%, Chi^2^=0.0, df=3 | |
| Random Effect Model | 0.59 | 0.01 |  | | [0.38,0.89] | -2.47 | | 0.072 | | I^2^=0.0%, Tau^2^=0.0 | |

Abbreviations: CA-AKI, contrast-associated acute kidney injury; CI, confidence interval; DF, degrees of freedom; NNT, number needed-to-treat; NR, not reported; RR, relative risk; SE, standard error.

**Table S18.** Quality assessment of Desch (2018)

| **Study question** | **Response**  **(yes/no/not clear/N/A)** | **How is the question addressed in the study?** |
| --- | --- | --- |
| **Was randomization carried out appropriately?** | Yes | Randomized in a 1:1 ratio to the treatment groups by permuted block randomization stratified by access site (radial/femoral) via a web-based system using a computer-generated list of random numbers. Randomization was performed before coronary angiography in the catheterization laboratory. |
| **Was the concealment of treatment allocation adequate?** | N/A | Due to the nature of the intervention, operators could not be blinded to treatment allocation. |
| **Were the groups similar at the outset of the study in terms of prognostic factors, for example, severity of disease?** | Yes | Baseline characteristics were well balanced between the two treatment groups. |
| **Were the care providers, participants, and outcome assessors blind to treatment allocation? If any of these people were not blinded, what might be the likely impact on the risk of**  **bias (for each outcome)?** | No | Due to the nature of the intervention, operators could not be blinded to treatment allocation which might have introduced bias. |
| **Were there any unexpected imbalances in drop-outs between groups? If so, were they explained or adjusted for?** | Yes | Figure 2 lists subject accountability and accounts for 2 drop-outs. Drop-out reasons are explained; n = 1 inclusion/exclusion violation, n = 1 contrast volume unavailable. |
| **Is there any evidence to suggest that the authors measured more outcomes than they reported?** | No | Outcomes provided and reported align with stated methods and planned endpoints. |
| **Did the analysis include an intention-to-treat analysis? If so, was this appropriate and were appropriate methods used to account for missing data?** | No | No details of intention-to-treat analysis reported. Based on Figure 2 and Section 3.2, 2 patients were excluded from the primary analysis (one due to a coronary anomaly and the other due to the primary endpoint data not being reported). |
| **Adapted from Centre for Reviews and Dissemination (2008) Systematic reviews. CRD’s guidance for undertaking reviews in health care. York: Centre for Reviews and Dissemination** | | |

**Table S19.** Quality assessment of Briguori (2020)

| **Study question** | **Response**  **(yes/no/not clear/N/A)** | **How is the question addressed in the study?** |
| --- | --- | --- |
| **Was the cohort recruited in an acceptable way?** | Yes | All patients with acute coronary syndrome who had urgent or immediate coronary angiography or angioplasty were evaluated. Diagnosis of acute coronary syndrome was established in accordance with guidelines. The study was approved by the local Ethics Committee. |
| **Was the exposure accurately measured to minimize bias?** | Yes | The associated Contrast Monitoring System (CMS, Osprey Medical, Minnesota, MN) displays contrast media volume injected (in ml), splitted in attempted, delivered, and saved (the last reported both as absolute value and as percentage vs. the total). |
| **Was the outcome accurately measured to minimize bias?** | Yes | Different statistical tests were used to analyze all types of outcomes. Rigorous adjustment for significant differences in the baseline characteristics of patients in the two groups was performed with propensity score matching to minimize bias. |
| **Have the authors identified all important confounding factors?** | Yes | Variables included in the logistic regression model to calculate the propensity score were (a) age; (b) sex; (c) Left ventricular ejection fraction; (d) Acute coronary syndrome type (ST-elevation myocardial infarction or non-ST-elevation myocardial infarction); (e) Mean systemic blood pressure; (f) Serum creatinine; (g) Glomerular filtration rate; (h) Diabetes mellitus; and (i) Peripheral artery disease. |
| **Have the authors taken account of the confounding factors in the design and/or analysis?** | Yes | Rigorous adjustment for significant differences in the baseline characteristics of patients in the two groups with propensity score matching performed. Appropriate statistical analyses were subsequently performed on each outcome. |
| **Was the follow-up of patients complete?** | Yes | Results were reported for all enrolled patients. 1 month follow-up was performed to assess major adverse events. |
| **How precise (for example, in terms of confidence interval and p values) are the results?** | Yes | Continuous variables are given as mean ± 1 standard deviation or median and first and third quartiles (Q1–Q3).  Categorical variables were reported as percentage and p-value.  Independent predictors of acute kidney injury were reported as odds ratio, 95% confidence interval, p-value, and bootstrap p. |
| **Adapted from Critical Appraisal Skills Program (CASP): Making sense of evidence 12 questions to help you make sense of a cohort study** | | |

**Table S20.** Quality assessment of Tajti (2019)

| **Study question** | **Response**  **(yes/no/not clear/N/A)** | **How is the question addressed in the study?** |
| --- | --- | --- |
| **Was the cohort recruited in an acceptable way?** | Yes | Consecutive enrolment of eligible patients undergoing chronic total occlusion percutaneous coronary intervention at Abbott Northwestern Hospital in Minneapolis, Minnesota (134 procedures amongst 130 patients). |
| **Was the exposure accurately measured to minimize bias?** | Yes | No evident bias in the way the exposure has been measured or classified. DyeVert system used amongst a proportion (30%) of all enrolled patients. |
| **Was the outcome accurately measured to minimize bias?** | Yes | All outcomes were defined and measured appropriately, including technical and procedural success, median contrast volume and volume savings, and complication rates. |
| **Have the authors identified all important confounding factors?** | N/A | Baseline clinical characteristics were reported as being similar across groups, as well as procedures performed, with no important confounding factors reported. |
| **Have the authors taken account of the confounding factors in the design and/or analysis?** | N/A | Appropriate statistical analyses have been performed to assess outcomes and to consider any confounding factors in the analysis, although none have been reported. |
| **Was the follow-up of patients complete?** | N/A | Study included in-hospital outcomes without long-term follow-up. |
| **How precise (for example, in terms of confidence interval and p values) are the results?** | Yes | 95% confidence intervals and p-values were reported for most outcomes, including differences in median contrast volume and complication rates. |
| **Adapted from Critical Appraisal Skills Program (CASP): Making sense of evidence 12 questions to help you make sense of a cohort study** | | |

**Table S21.** Quality assessment of Zimin (2020)

| **Study question** | **Response**  **(yes/no/not clear/N/A)** | **How is the question addressed in the study?** |
| --- | --- | --- |
| **Was the cohort recruited in an acceptable way?** | Yes | The Baptist Health Lexington Institutional Review Board approved this study, and all subjects provided written informed consent. Study conducted at 2 research centers, one which enrolled all participants and the other which performed imaging procedures. |
| **Was the exposure accurately measured to minimize bias?** | Yes | Data collected post-procedurally included characteristics of image quality (including clear image length, clear stent length, and clear region of interest) assessed by the imaging core laboratory. Intervention performed amongst all patients undergoing optical coherence tomography procedures, and exposure appears to have been objectively assessed. |
| **Was the outcome accurately measured to minimize bias?** | Yes | Quality of angiographic and optical coherence tomography images was evaluated both subjectively in real time by the physician operators at the conclusion of each procedure and subsequently by the imaging core laboratory using objective data. No evidence of bias in outcome measurement. |
| **Have the authors identified all important confounding factors?** | N/A | No confounding factors reported. |
| **Have the authors taken account of the confounding factors in the design and/or analysis?** | N/A | Appropriate statistical analyses performed to assess outcomes, but no confounding factors reported. |
| **Was the follow-up of patients complete?** | N/A | There was no clinical follow-up beyond the end of optical coherence tomography acquisition. |
| **How precise (for example, in terms of confidence interval and p values) are the results?** | Yes | Standard deviations along with p-values were reported for all outcomes. Mean values as well as 97.5% confidence intervals also reported. |
| **Adapted from Critical Appraisal Skills Program (CASP): Making sense of evidence 12 questions to help you make sense of a cohort study** | | |

**Table S22.** Quality assessment of Sapontis (2017)

| **Study question** | **Response**  **(yes/no/not clear/N/A)** | **How is the question addressed in the study?** |
| --- | --- | --- |
| **Was the cohort recruited in an acceptable way?** | Yes | Consecutive enrolment of eligible subjects occurred at both sites with completing of informed consent. The study was approved by a local ethics committee. |
| **Was the exposure accurately measured to minimize bias?** | Yes | The contrast monitoring system was used to electronically display the injected amount of contrast volume in ml. The contrast monitoring system has been shown to accurately calculate contrast volume used when compared to a standard manual method of contrast accounting and is superior to common practice of estimated use by a physician. No evidence of bias in measurement of the exposure. |
| **Was the outcome accurately measured to minimize bias?** | Yes | Contrast saved was determined by subtracting the volume delivered to the patient from the cumulative volume attempted to be injected into the patient. The cumulative volume attempted to be injected was provided by the digital read out on the contrast monitoring system device. To calculate this endpoint, the percent of attempted injection volume saved was determined by calculating each subjects’ percentage contrast media savings and then calculating the mean of all values. No evidence of bias in outcome measurement. |
| **Have the authors identified all important confounding factors?** | N/A | No confounding factors reported. |
| **Have the authors taken account of the confounding factors in the design and/or analysis?** | N/A | Appropriate statistical analyses performed to assess outcomes, but no confounding factors reported. |
| **Was the follow-up of patients complete?** | N/A | Only short-term outcomes assessed, and no follow-up reported. |
| **How precise (for example, in terms of confidence interval and p values) are the results?** | Yes | Standard deviations along with minimum and maximums were reported for all outcomes. For the primary endpoint, the mean percent volume saved, and p-value, are reported. |
| **Adapted from Critical Appraisal Skills Program (CASP): Making sense of evidence 12 questions to help you make sense of a cohort study** | | |

**Table S23.** Quality assessment of Corcione (2017)

| **Study question** | **Response**  **(yes/no/not clear/N/A)** | **How is the question addressed in the study?** |
| --- | --- | --- |
| **Was the cohort recruited in an acceptable way?** | Yes | Patients provided written, informed consent to participate in the study, and the institutional clinical database from which the study was performed was ethics-committee approved. |
| **Was the exposure accurately measured to minimize bias?** | Yes | The DyeVert Plus system was connected to the contrast source and the manifold, enabling the diversion of excess contrast during injections through the pressure-compensating valve. The intervention was applied as per routine clinical practice, with no evidence of bias in exposure measurement. |
| **Was the outcome accurately measured to minimize bias?** | Yes | Contrast volume administered to the patient, as calculated and displayed by the DyeVert Plus system, was compared with manual measurements at the end of the procedure. Manual measurements were determined by a labour-intensive process of emptying all contrast from tubing used in the procedure into metered vials and subtracting this amount from the starting volume in the bottle. No evidence of bias in outcome measurement. |
| **Have the authors identified all important confounding factors?** | N/A | Single site, small sample size, short-term and no control group. No confounding factors have been reported. |
| **Have the authors taken account of the confounding factors in the design and/or analysis?** | N/A | Appropriate statistical analyses performed to assess outcomes, but no confounding factors reported. |
| **Was the follow-up of patients complete?** | N/A | No follow-up beyond assessment of the immediate study outcomes performed. |
| **How precise (for example, in terms of confidence interval and p values) are the results?** | Yes | Inferential analysis for mean and median values with p-values and 95% confidence intervals were obtained from percentile bootstrapping (1,000 samples). |
| **Adapted from Critical Appraisal Skills Program (CASP): Making sense of evidence 12 questions to help you make sense of a cohort study** | | |

**Table S24.** Quality assessment of Gurm (2019)

| **Study question** | **Response**  **(yes/no/not clear/N/A)** | **How is the question addressed in the study?** |
| --- | --- | --- |
| **Was the cohort recruited in an acceptable way?** | Yes | Local institutional review boards approved this study and all subjects provided written informed consent. Patients were undergoing diagnostic coronary angiography and/or percutaneous coronary interventional procedures performed with manual injections, at eight centers. |
| **Was the exposure accurately measured to minimize bias?** | Yes | The DyeVert Plus System interfaces with standard manifold systems to provide real-time contrast monitoring and reduce the amount of contrast used in catheterization procedures while maintaining fluoroscopic image quality. The intervention was utilised amongst patients undergoing the outlined procedures, with no evidence of bias in measuring the exposure. |
| **Was the outcome accurately measured to minimize bias?** | Yes | At the end of the procedure, the contrast monitoring wireless displays total procedure contrast volume used (mL, actual contrast media volume delivered to the patient), % of physician-specified threshold, total procedure contrast volume saved (mL), and % contrast saved. Outcomes were measured appropriately, with no evidence of bias. |
| **Have the authors identified all important confounding factors?** | N/A | Single-arm study with no control group. No confounding factors have been reported. |
| **Have the authors taken account of the confounding factors in the design and/or analysis?** | N/A | Appropriate statistical analyses performed to assess outcomes. Criteria applied to determine those patients evaluable for primary endpoint analysis, but no confounding factors reported. |
| **Was the follow-up of patients complete?** | N/A | Details of patient follow-up not reported, but this would appear to be due to the short-term nature of the outcomes included in the analysis. |
| **How precise (for example, in terms of confidence interval and p values) are the results?** | Yes | Standard deviations along with minimum and maximum values were reported for the outcomes. 95% confidence interval and p-value were reported for the primary endpoint. |
| **Adapted from Critical Appraisal Skills Program (CASP): Making sense of evidence 12 questions to help you make sense of a cohort study** | | |

**Table S25.** Quality assessment of Bruno (2019)

| **Study question** | **Response**  **(yes/no/not clear/N/A)** | **How is the question addressed in the study?** |
| --- | --- | --- |
| **Was the cohort recruited in an acceptable way?** | Yes | They performed this retrospective pilot study using their ethics committee-approved institutional clinical database in two consecutive days in November 2018. All patients who underwent invasive coronary angiography procedures with the DyeVert System were included. |
| **Was the exposure accurately measured to minimize bias?** | Yes | All procedures were performed under conditions of standard clinical practice by four different physicians. No adaptations to routine diagnostic and guiding catheters, guide wires, balloons, or stents were made, and there was no evidence of bias in measuring the exposure. |
| **Was the outcome accurately measured to minimize bias?** | Yes | Study measured the volume of contrast delivered to the patient, the attempted amount delivered, and the volume saved. There was no evidence of bias in measuring the outcome. |
| **Have the authors identified all important confounding factors?** | N/A | Single site, small sample size, short-term and no control group. No confounding factors have been reported. |
| **Have the authors taken account of the confounding factors in the design and/or analysis?** | N/A | No confounding factors reported or considered in the analysis of outcomes. |
| **Was the follow-up of patients complete?** | N/A | Only short-term outcomes were assessed, and therefore patient follow-up was not applicable. |
| **How precise (for example, in terms of confidence interval and p values) are the results?** | No | Mean and standard deviation values have been reported. However, p-value and 95% confidence intervals not reported for outcomes. |
| **Adapted from Critical Appraisal Skills Program (CASP): Making sense of evidence 12 questions to help you make sense of a cohort study** | | |

**Table S26.** Quality assessment of Bath (2019)

| **Study question** | **Response**  **(yes/no/not clear/N/A)** | **How is the question addressed in the study?** |
| --- | --- | --- |
| **Was randomization carried out appropriately?** | Not clear | Insufficient details of randomization provided to assess. |
| **Was the concealment of treatment allocation adequate?** | N/A | Due to the nature of the intervention, operators could not be blinded to treatment allocation. |
| **Were the groups similar at the outset of the study in terms of prognostic factors, for example, severity of disease?** | Not clear | Baseline characteristics not reported, other than to indicate that all patients in the analysis were at increased risk of contrast induced nephropathy. |
| **Were the care providers, participants, and outcome assessors blind to treatment allocation? If any of these people were not blinded, what might be the likely impact on the risk of**  **bias (for each outcome)?** | No | Due to the nature of the intervention, operators could not be blinded to treatment allocation which might have introduced bias. |
| **Were there any unexpected imbalances in drop-outs between groups? If so, were they explained or adjusted for?** | Not clear | No details of drop-outs reported. |
| **Is there any evidence to suggest that the authors measured more outcomes than they reported?** | No | The outcomes reported in the methods of the analysis have been appropriately described in the results. |
| **Did the analysis include an intention-to-treat analysis? If so, was this appropriate and were appropriate methods used to account for missing data?** | Not clear | Insufficient detail reported to determine if an intention-to-treat analysis was performed. |
| **Adapted from Centre for Reviews and Dissemination (2008) Systematic reviews. CRD’s guidance for undertaking reviews in health care. York: Centre for Reviews and Dissemination** | | |

**Table S27.** Quality assessment of Sattar (2018)

| **Study question** | **Response**  **(yes/no/not clear/N/A)** | **How is the question addressed in the study?** |
| --- | --- | --- |
| **Was the cohort recruited in an acceptable way?** | Yes | Use of the device, and inclusion of patients, at the treating cardiologist’s discretion. Those eligible were patients undergoing cardiac catheterization with coronary interventions with chronic kidney disease defined as having a glomerular filtration rate < 60 by MRDR calculation method  and/or Serum Creatinine >1.5. |
| **Was the exposure accurately measured to minimize bias?** | Yes | Patients received the usual pre and post procedural hydration and utilised the DyeVert System during their procedure, which was performed as per clinical practice. No evidence of bias in measuring the exposure. |
| **Was the outcome accurately measured to minimize bias?** | Yes | Patients incurring acute kidney injury were defined as an absolute increase of ≥ 0.3mg/dL or a relative increase of 50% in serum creatinine. No evidence of bias in measuring the outcome. |
| **Have the authors identified all important confounding factors?** | Not clear | No details of confounding factors reported, but unclear if these were present. |
| **Have the authors taken account of the confounding factors in the design and/or analysis?** | Not clear | Unclear if confounding factors were present, but appropriate methods performed in the analysis of outcomes. The authors do report that bias may have been present in the analysis due to general awareness of contrast levels and acute kidney injury. |
| **Was the follow-up of patients complete?** | N/A | No patient follow-up reported. Short-term outcomes were assessed. |
| **How precise (for example, in terms of confidence interval and p values) are the results?** | Yes | Mean values, confidence intervals, p-values, and odds ratios all reported. |
| **Adapted from Critical Appraisal Skills Program (CASP): Making sense of evidence 12 questions to help you make sense of a cohort study** | | |

**Table S28.** Quality assessment of Kutschman (2019)

| **Study question** | **Response**  **(yes/no/not clear/N/A)** | **How is the question addressed in the study?** |
| --- | --- | --- |
| **Was the cohort recruited in an acceptable way?** | Yes | Protocol and DyeVert system use implemented amongst patients with chronic kidney disease, with those patients already on dialysis omitted. Pre-procedural risk assessment was performed as well as pre and post procedural hydration. |
| **Was the exposure accurately measured to minimize bias?** | Yes | DyeVert System implemented among patients undergoing standard procedures. No evidence of bias in measuring the exposure. |
| **Was the outcome accurately measured to minimize bias?** | Yes | Primary outcome was % CM volume savings and this appeared to be appropriately measured. No evidence of bias in measurement. |
| **Have the authors identified all important confounding factors?** | Not clear | No details of confounding factors reported, but unclear if these were present. |
| **Have the authors taken account of the confounding factors in the design and/or analysis?** | Not clear | Unclear if confounding factors were present, but appropriate methods performed in the analysis of outcomes. |
| **Was the follow-up of patients complete?** | N/A | No patient follow-up reported. |
| **How precise (for example, in terms of confidence interval and p values) are the results?** | No | Results reported in terms of mean volume per case, overall savings, relative reduction expressed as a percentage, number needed to treat to avoid an acute kidney injury event, and cost savings. However, no details of statistical significance of contrast media savings (p-values) provided. |
| **Adapted from Critical Appraisal Skills Program (CASP): Making sense of evidence 12 questions to help you make sense of a cohort study** | | |

**Table S29.** Quality assessment of Kutschman (2019)

| **Study question** | **Response**  **(yes/no/not clear/N/A)** | **How is the question addressed in the study?** |
| --- | --- | --- |
| **Was the cohort recruited in an acceptable way?** | Yes | Use of the intervention mandated for all inpatient procedures, and for outpatient procedures with estimated glomerular filtration rate < 60, following initiation of protocol. Standardized  pre-procedure patient risk assessment practices were performed. |
| **Was the exposure accurately measured to minimize bias?** | Yes | DyeVert System implemented amongst patients undergoing standard procedures. No evidence of bias in measuring the exposure. |
| **Was the outcome accurately measured to minimize bias?** | Yes | Change in acute kidney injury rate following use of the intervention appears to have been appropriately measured. No evidence of bias. |
| **Have the authors identified all important confounding factors?** | Not clear | No details of confounding factors reported, but unclear if these were present. |
| **Have the authors taken account of the confounding factors in the design and/or analysis?** | Not clear | Unclear if confounding factors were present, but appropriate methods performed in the analysis of outcomes. |
| **Was the follow-up of patients complete?** | N/A | No patient follow-up reported. |
| **How precise (for example, in terms of confidence interval and p values) are the results?** | No | Results report % change in acute kidney injury rate, mean contrast media volume savings and risk reduction in acute kidney injury rate expressed as a percentage. No confidence intervals or p-values reported. |
| **Adapted from Critical Appraisal Skills Program (CASP): Making sense of evidence 12 questions to help you make sense of a cohort study** | | |

**Table S30.** Quality assessment of Bunney (2019)

| **Study question** | **Response**  **(yes/no/not clear/N/A)** | **How is the question addressed in the study?** |
| --- | --- | --- |
| **Was the cohort recruited in an acceptable way?** | Yes | Patients undergoing percutaneous coronary intervention using the intervention in the hospital over a period of 3 years were identified and included. Data from these patients compared to data from patients who didn’t receive the intervention. |
| **Was the exposure accurately measured to minimize bias?** | Yes | Intervention implemented amongst patients undergoing standard procedures. No evidence of bias in measuring the exposure. |
| **Was the outcome accurately measured to minimize bias?** | Yes | Study looked at association between use of the intervention and clinical characteristics, acute kidney injury rate etc. No evidence of bias in measuring the outcomes. |
| **Have the authors identified all important confounding factors?** | Yes | Baseline clinical characteristics were reported as being similar across groups, although authors report that more complex percutaneous coronary interventions were performed in the DyeVert group. |
| **Have the authors taken account of the confounding factors in the design and/or analysis?** | Not clear | It is unclear whether any analyses were performed in relation to outcome assessment to account for the more complex procedures performed in the DyeVert group. |
| **Was the follow-up of patients complete?** | N/A | No patient follow-up reported. It appears that only short-term outcomes were assessed. |
| **How precise (for example, in terms of confidence interval and p values) are the results?** | No | The reporting of percentages was appropriate given the outcomes being assessed. However, confidence intervals not reported. |
| **Adapted from Critical Appraisal Skills Program (CASP): Making sense of evidence 12 questions to help you make sense of a cohort study** | | |

**Table S31.** Quality assessment of Turner/Tucker (2020)

| **Study question** | **Response**  **(yes/no/not clear/N/A)** | **How is the question addressed in the study?** |
| --- | --- | --- |
| **Was the cohort recruited in an acceptable way?** | Yes | Intervention used as part of a quality-improvement protocol, amongst patients with estimated glomerular filtration rate < 60 or creatinine > 1.5 or ST-elevation myocardial infarction. |
| **Was the exposure accurately measured to minimize bias?** | Yes | Intervention implemented amongst patients undergoing angiography procedures, carried out according to standard practice. No evidence of bias in measurement of the exposure. |
| **Was the outcome accurately measured to minimize bias?** | Yes | Change in acute kidney injury rate was the primary outcome, with contrast-induced acute kidney injury defined as an increase in serum creatinine of ≥ 0.3mg/dl or ≥ 50% within ≤ 48 hours, tracked via the American College of Cardiology’s National Cardiovascular Data Registry CathPCI Registry as the risk-adjusted contrast-induced acute kidney injury metric. No evidence of bias in measurement of the outcome. |
| **Have the authors identified all important confounding factors?** | Not clear | No details of confounding factors reported, but unclear if these were present. |
| **Have the authors taken account of the confounding factors in the design and/or analysis?** | Not clear | Unclear if confounding factors were present, but appropriate methods performed in the analysis of outcomes. |
| **Was the follow-up of patients complete?** | N/A | No patient follow-up reported. Outcomes assessed did not require long-term follow-up. |
| **How precise (for example, in terms of confidence interval and p values) are the results?** | No | Absolute % reduction, relative risk, and cost savings reported. However, no confidence intervals reported. |
| **Adapted from Critical Appraisal Skills Program (CASP): Making sense of evidence 12 questions to help you make sense of a cohort study** | | |

**Table S32.** Quality assessment of Cameron (2020)

| **Study question** | **Response**  **(yes/no/not clear/N/A)** | **How is the question addressed in the study?** |
| --- | --- | --- |
| **Was the cohort recruited in an acceptable way?** | Yes | Implementation of a quality improvement initiative amongst patients undergoing coronary angiography procedures between January 2018 and March 2019. |
| **Was the exposure accurately measured to minimize bias?** | Yes | Intervention implemented amongst patients undergoing procedures at a hospital cardiac catheterization laboratory, carried out according to standard practice. No evidence of bias in measurement of the exposure. |
| **Was the outcome accurately measured to minimize bias?** | Yes | Incidence of contrast-induced acute kidney injury, following introduction of the initiative compared to prior to introduction. No evidence of bias in measurement of the outcome. |
| **Have the authors identified all important confounding factors?** | Not clear | No details of confounding factors reported, but unclear if these were present. |
| **Have the authors taken account of the confounding factors in the design and/or analysis?** | Not clear | Unclear if confounding factors were present, but appropriate methods performed in the analysis of outcomes. |
| **Was the follow-up of patients complete?** | Yes | Outcomes were reported 9 months after implementation of the protocol. No evidence of data omissions. |
| **How precise (for example, in terms of confidence interval and p values) are the results?** | No | Only % change in rate of contrast-induced acute kidney injury following implementation of the protocol reported. 95% confidence intervals not reported. |
| **Adapted from Critical Appraisal Skills Program (CASP): Making sense of evidence 12 questions to help you make sense of a cohort study** | | |

**Table S33.** Quality assessment of Rao (2019)

| **Study question** | **Response**  **(yes/no/not clear/N/A)** | **How is the question addressed in the study?** |
| --- | --- | --- |
| **Was the cohort recruited in an acceptable way?** | Not clear | Assessment of use of the intervention amongst patients with critical limb ischaemia, undergoing highly complex peripheral vascular interventions. However, no details of patient consent or enrolment provided. |
| **Was the exposure accurately measured to minimize bias?** | Yes | Intervention implemented amongst patients undergoing highly complex peripheral vascular interventions. No evidence of bias in measurement of the exposure. |
| **Was the outcome accurately measured to minimize bias?** | Yes | Outcomes assessed included contrast media volume/estimated glomerular filtration rate, mean contrast media volume, renal function, and image quality. No evidence of bias in measurement of outcomes. |
| **Have the authors identified all important confounding factors?** | Not clear | No details of confounding factors reported, but unclear if these were present. Unlikely to be relevant given small sample size and similarity of procedures performed. |
| **Have the authors taken account of the confounding factors in the design and/or analysis?** | Not clear | Unclear if confounding factors were present, but appropriate methods performed in the analysis of outcomes. |
| **Was the follow-up of patients complete?** | N/A | No patient follow-up reported. |
| **How precise (for example, in terms of confidence interval and p values) are the results?** | Yes | Mean values and ranges reported, which were appropriate given the outcomes assessed. |
| **Adapted from Critical Appraisal Skills Program (CASP): Making sense of evidence 12 questions to help you make sense of a cohort study** | | |

**Table S34.** Quality assessment of Amoroso (2020)

| **Study question** | **Response**  **(yes/no/not clear/N/A)** | **How is the question addressed in the study?** |
| --- | --- | --- |
| **Was the cohort recruited in an acceptable way?** | Not clear | Assessment of use of the intervention amongst patients undergoing coronary angiography procedures at three hospitals between September – November 2018. However, no details of patient consent or enrolment provided. |
| **Was the exposure accurately measured to minimize bias?** | Yes | All patient care and automated contrast injector system settings were completed per standard of care and there were no study-induced procedures. Intervention implemented as part of procedures performed. |
| **Was the outcome accurately measured to minimize bias?** | Not clear | Outcomes measured included contrast media volume savings and user satisfaction. No evidence of bias in outcome measurements, however insufficient details of physician satisfaction survey provided to evaluate. |
| **Have the authors identified all important confounding factors?** | Not clear | No details of confounding factors reported, but unclear if these were present. Unlikely to be relevant given small sample size and similarity of procedures performed. |
| **Have the authors taken account of the confounding factors in the design and/or analysis?** | Not clear | Unclear if confounding factors were present, but appropriate methods performed in the analysis of outcomes. |
| **Was the follow-up of patients complete?** | N/A | No patient follow-up reported. |
| **How precise (for example, in terms of confidence interval and p values) are the results?** | No | Mean values and ranges for contrast media volume savings reported, with the % of respondents reporting satisfaction also presented. However, no p-values or details of statistical significance reported. |
| **Adapted from Critical Appraisal Skills Program (CASP): Making sense of evidence 12 questions to help you make sense of a cohort study** | | |

# Supplemental Figures

**Figure S1.** Meta-analysis: DyeVert group Proportion of attempted contrast media volume diverted by the DyeVert System (%) in observational studies, DyeVert Products Used with Manual CM Injection Systems


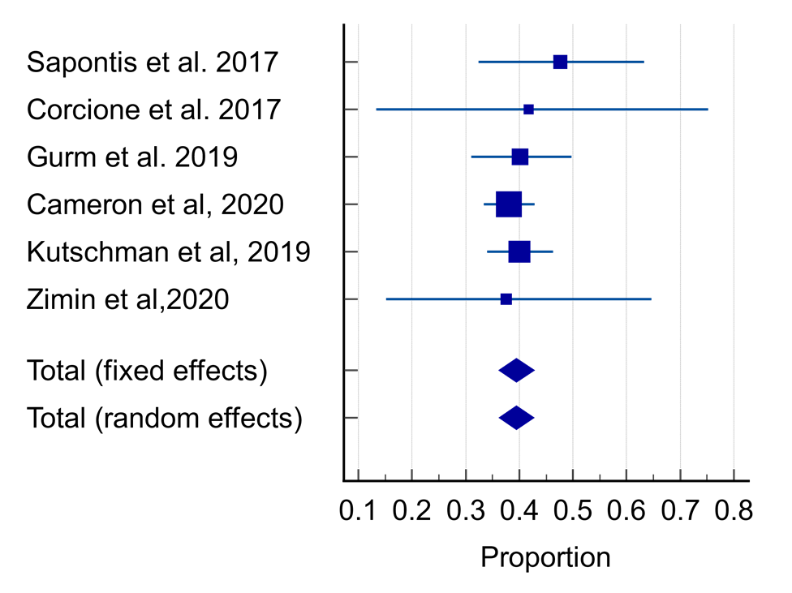


Figure S2. Meta-analysis: Forest plot of pooled estimate of contrast-associated acute kidney injury incidence in DyeVert group

A) Five Studies, Primary Analysis


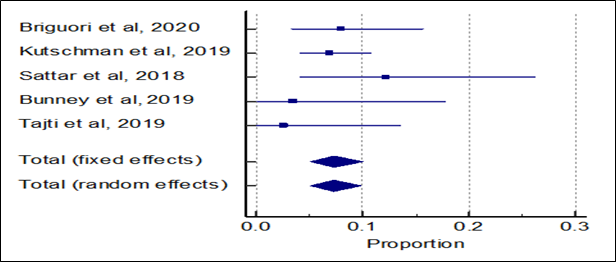


B) Four Studies, Excluding Tajti (2019)


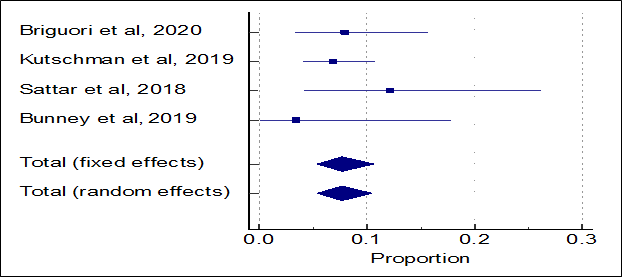


Figure S3. Meta-analysis: Forest plot of pooled estimate of contrast-associated acute kidney injury incidence in control group

A) Five Studies, Primary Analysis


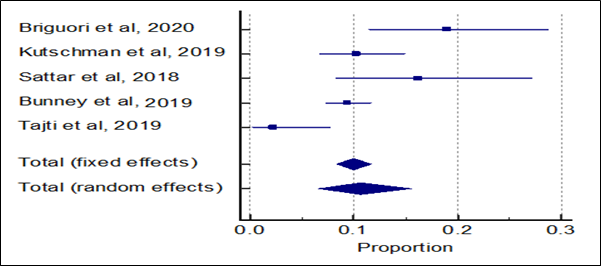


B) Four Studies, Excluding Tajti (2019)


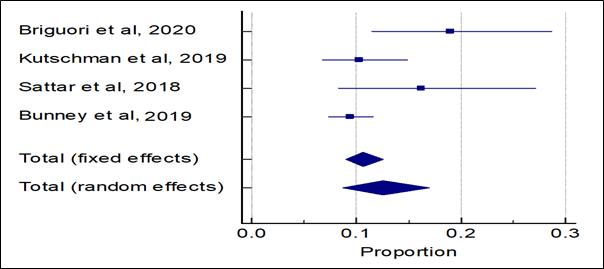


**Figure S4.**  Meta-analysis: Forest plot of relative risk and absolute risk reduction of contrast-associated acute kidney injury in the DyeVert group versus Control group

A.


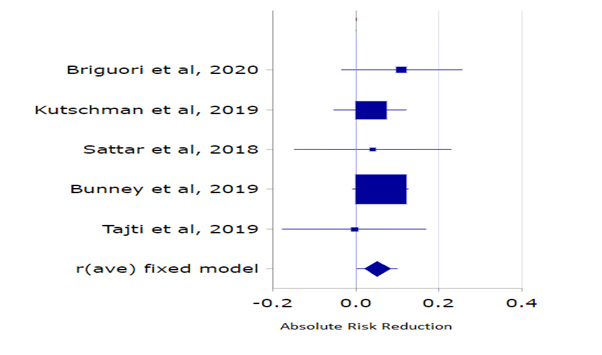


B.


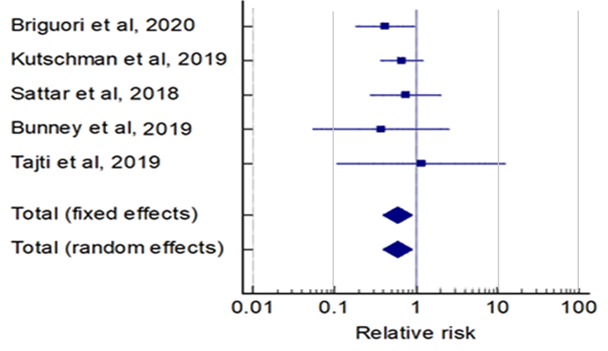


C.


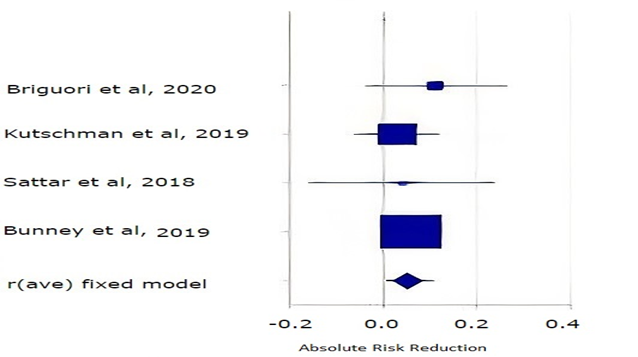


D.


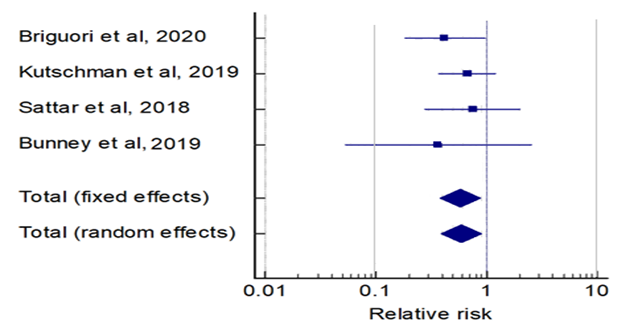


Five studies: A) Absolute risk reduction; B) Relative risk.
Four studies, excluding Tajti (2019): C) Absolute risk reduction; D) Relative risk.

**REFERENCES:**

**Amoroso** G, Christian J, Christopher A. First European experience using a novel contrast reduction system during coronary angiography with automated contrast injection. Abstract accepted for presentation at EuroPCR 2020; May 2020 (cancelled due to COVID); Paris, France. *EuroIntervention*. 2020;16(AC)(suppl):Euro20A-POS426. Accessed at: https://eurointervention.pcronline.com/news/pcr-e-course-abstract-book-2020.

**Bath** A, Bobba K, Gautam S, Gupta V. Use of DyeVert Plus to reduce contrast exposure in high-risk patients undergoing coronary angiography. Abstract. *J Am Coll Cardiol*. 2019;73(suppl 1):1193. doi:10.1016/S0735-1097(19)31800-5

**Briguori** C, Golino M, Porchetta N, Scarpelli M, De Micco F, Rubino C, Focaccio A, Signoriello G. Impact of a contrast media volume control device on acute kidney injury rate in patients with acute coronary syndrome. *Catheter Cardiovasc Interv*. Published online July 18, 2020. 2021;98:76- 84. doi:10.1002/ccd.29136

**Bruno** RR, Nia AM, Wolff G, Erkens R, Kelm M, Westenfeld R, Jung C. Early clinical experiences with a novel contrast volume reduction system during invasive coronary angiography. *Int J Cardiol Heart Vasc*. 2019;23:100377. doi:10.1016/j.ijcha.2019.100377

**Bunney** R, Saenger E, Shah, C, Harris S, Hill-Herrera K, Leopold D, Caldera S, Smith R, Phillips J, Prasad A. Contemporary use of contrast dye reduction technology in a tertiary academic hospital: patient characteristics and acute kidney injury outcomes following percutaneous coronary interventions. Poster presented at: American College of Cardiology (ACC) Quality Summit; March 2019; New Orleans, LA. Poster 2018-063. https://cvquality.acc.org/docs/default-source/quality-poster-awards-2019/2018-063_bunney-robert_rn.pdf?sfvrsn=582f86bf_2.

**Cameron** A, Espinosa TJ. Reducing contrast-induced acute kidney injury in a cardiac catherization laboratory: a quality improvement initiative. Eposter abstract presented at: Society for Cardiac Angiography & Interventions Scientific Sessions Virtual Conference. https://virtual2020.scai.org/p/46024 *Catheter Cardiovasc Interv*. 2020;95(suppl 2):I-34. doi:10.1002/ccd.28864

**Centre for Reviews and Dissemination**. Systematic Reviews – CRDs guidance for undertaking reviews in healthcare. York.ac.uk. 2009. [online] Available at: <https://www.york.ac.uk/media/crd/Systematic_Reviews.pdf> [Accessed 1 March 2022].

**Corcione** N, Biondi-Zoccai G, Ferraro P, Messina S, Maresca G, Avellino R, Napolitano G, Cavarretta E, Giordano A. Contrast minimization with the new-generation DyeVert Plus System for contrast reduction and real-time monitoring during coronary and peripheral procedures: first experience. *J Invasive Cardiol*. 2017;29:259-262. PMID: 28756419

**Critical Appraisal Skills Programme**. CASP (Cohort Study) Checklist 2018. [online] Available at: https://casp-uk.net/wp-content/uploads/2018/01/CASP-Cohort-Study-Checklist_2018.pdf [Accessed: 01/03/2022].

**Desch** S, Fuernau G, Pöss J, Meyer-Saraei R, Saad M, Eitel I, Thiele H, de Waha S. Impact of a novel contrast reduction system on contrast savings in coronary angiography - the DyeVert randomised controlled trial. *Int J Cardiol*. 2018;257:50-53. doi:10.1016/j.ijcard.2017.12.107

**Gurm** HS, Mavromatis K, Bertolet B, Kereiakes DJ, Amin AP, Shah AP, Hanzel GS, Rao S, Thomas JL, Kumar G. Minimizing radiographic contrast administration during coronary angiography using a novel contrast reduction system: a multicenter observational study of the DyeVert™ plus contrast reduction system. *Catheter Cardiovasc Interv*. 2019;93:1228-1235. doi:10.1002/ccd.27935

**Kutschman** R. Clinical and economic outcomes of a comprehensive clinical quality initiative for reducing acute kidney injury in chronic kidney disease patients undergoing coronary angiography. Abstract published at the Thirty First Annual Transcatheter Cardiovascular Therapeutics Symposium, held September 25-29, 2019, San Francisco, CA. *J Am Coll Cardiol*. 2019;74(suppl):B605. Accessed at https://www.sciencedirect.com/journal/journal-of-the-american-college-of-cardiology/vol/74/issue/13/suppl/S.

**Kutschman** R, Davison L, Beyer J. Comprehensive clinical quality initiative for reducing acute kidney injury in at-risk patients undergoing diagnostic coronary angiogram and/or percutaneous coronary interventions. Poster presented at: Society for Cardiac Angiography & Interventions 2019 Scientific Sessions; May 2019; Las Vegas, NV. Accessed January 20, 2021. Accessed at: https://scai.confex.com/scai/2019/webprogram/Paper2030.html.

**Rao** S. DyeVert Plus Contrast Reduction System use in patients undergoing highly complex peripheral vascular interventions. Poster presented at: International Symposium on Endovascular Therapies (ISET); January 2019; Hollywood, CA. *J Vasc Interv Radiol*. 2019;30:e16. doi:10.1016/j.jvir.2018.11.033. Accessed at: https://www.jvir.org/article/S1051-0443(18)31737-8/pdf.

**Sapontis** J, Barron G, Seneviratne S, Fuernau G, Eitel I, Ledwoch J, Thiele H, Saad M, de Waha S, Poess J, et al. A first in human evaluation of a novel contrast media saving device. *Catheter Cardiovasc Interv*. 2017;90:928-934. doi:10.1002/ccd.27033

**Sattar** A, Schnatz R, Darby M, El-Hamdani M. Impact of using DyeVert PLUS on incidence of acute kidney injury after cardiac catheterization with coronary interventions in high-risk patients. Poster presented at: American College of Cardiology Annual Meeting; April 2018; Charleston, WV. Poster 88-89. Accessed at: https://jcesom.marshall.edu/media/56993/2018-abstracts.pdf.

**Tajti** P, Xenogiannis I, Hall A, Burke MN, Chavez I, Garcia S, Gössl M, Mooney M, Poulose A, Sorajja P, et al. Use of the DyeVert system in chronic total occlusion percutaneous coronary intervention. *J Invasive Cardiol*. 2019;31:253-259. PMID:31478890

**Turner** C, Tucker PA. Real-world impact of a quality improvement program for AKI prevention in the cardiac cath lab. Abstract at: Society for Cardiac Angiography & Interventions Scientific Sessions Virtual Conference. *Catheter Cardiovasc Interv*. 2020;95(suppl 2):S112-S3.

**Zimin** VN, Jones MR, Richmond IT, Durieux JC, Alaiti AM, Pereira G, Vergara-Martel A, Pizzato PE, Zago EI, Dallan LA, et al. A feasibility study of the DyeVert™ plus contrast reduction system to reduce contrast media volumes in percutaneous coronary procedures using optical coherence tomography. *Cardiovasc Revasc Med*. Published online October 3, 2020. 2021;30:40-46. doi:10.1016/j.carrev.2020.09.040
